# G-MDSCs promote aging-related cardiac fibrosis by activating myofibroblasts and preventing senescence

**DOI:** 10.1038/s41419-021-03874-7

**Published:** 2021-06-08

**Authors:** Shu-Ning Sun, Shi-Hao Ni, Yue Li, Xin Liu, Jian-Ping Deng, Zi-Xin Chen, Huan Li, Wen-Jun Feng, Yu-Sheng Huang, Da-Nian Li, Shao-Xiang Xian, Zhong-Qi Yang, Ling-Jun Wang, Lu Lu

**Affiliations:** 1 The First Affiliated Hospital, Guangzhou University of Chinese Medicine, Guangzhou, 510407 China; 2 Lingnan Medical Research Center, Guangzhou University of Chinese Medicine, Guangzhou, 510407 China; 3 Key Laboratory of Chronic Heart Failure, Guangzhou University of Chinese Medicine, Guangzhou, 510407 China

**Keywords:** Senescence, Cardiovascular diseases

## Abstract

Aging is one of the most prominent risk factors for heart failure. Myeloid-derived suppressor cells (MDSCs) accumulate in aged tissue and have been confirmed to be associated with various aging-related diseases. However, the role of MDSCs in the aging heart remains unknown. Through RNA-seq and biochemical approaches, we found that granulocytic MDSCs (G-MDSCs) accumulated significantly in the aging heart compared with monocytic MDSCs (M-MDSCs). Therefore, we explored the effects of G-MDSCs on the aging heart. We found that the adoptive transfer of G-MDSCs of aging mice to young hearts resulted in cardiac diastolic dysfunction by inducing cardiac fibrosis, similar to that in aging hearts. S100A8/A9 derived from G-MDSCs induced inflammatory phenotypes and increased the osteopontin (OPN) level in fibroblasts. The upregulation of fibroblast growth factor 2 (FGF2) expression in fibroblasts mediated by G-MDSCs promoted antisenescence and antiapoptotic phenotypes of fibroblasts. SOX9 is the downstream gene of FGF2 and is required for FGF2-mediated and G-MDSC-mediated profibrotic effects. Interestingly, both FGF2 levels and SOX9 levels were upregulated in fibroblasts but not in G-MDSCs and were independent of S100A8/9. Therefore, a novel FGF2-SOX9 signaling axis that regulates fibroblast self-renewal and antiapoptotic phenotypes was identified. Our study revealed the mechanism by which G-MDSCs promote cardiac fibrosis via the secretion of S100A8/A9 and the regulation of FGF2-SOX9 signaling in fibroblasts during aging.

## Introduction

Aging is known to be one of the most important cardiovascular risk factors^1^. The proportion of individuals with diastolic dysfunction or even heart failure significantly increases with advancing age^2^. Aging induces cardiac biological changes, resulting in hypertrophy, a decline in diastolic function, or even heart failure^3,4^. Functionally, aging-related heart disease is characterized by heart failure with preserved ejection fraction (HFpEF) caused by cardiac fibrosis, which results from changes in the cardiac microenvironment, including senescence, collagen deposition, inflammation and cell death, and has been shown to have clinical importance^5^. Thus, the mechanisms of aging-related cardiac fibrosis deserve further exploration.

Myeloid-derived suppressor cells (MDSCs) are a heterogeneous population of immature myeloid cells that suppress innate and adaptive immunity^6,7^. MDSCs consist of two large groups of cells: granulocytic MDSCs (G-MDSCs) are phenotypically and morphologically similar to neutrophils, and monocytic MDSCs (M-MDSCs) are similar to monocytes^8^. Previous studies have demonstrated that MDSCs are most commonly identified by LOX or CD33 expression in humans, whereas in mice, MDSCs were found to be myeloid-derived cells (Gr1 + CD11b + )^9,10^. Initially, MDSCs were identified as a major obstacle for natural antitumor immunities and were found in many other abnormal conditions, such as autoimmunity, infection, and diabetes^10^. MDSCs act as a double-edged sword in different disorders. Although a series of studies have verified the expansion of MDSCs with age^11,12^, the effects of these cells on aging hearts are still unclear. In this study, we sought to explore the role of MDSCs in the aging heart and the mechanism of the G-MDSC profibrotic function.

## Results

### The G-MDSC levels increase with advancing age

**Fig. 1:**
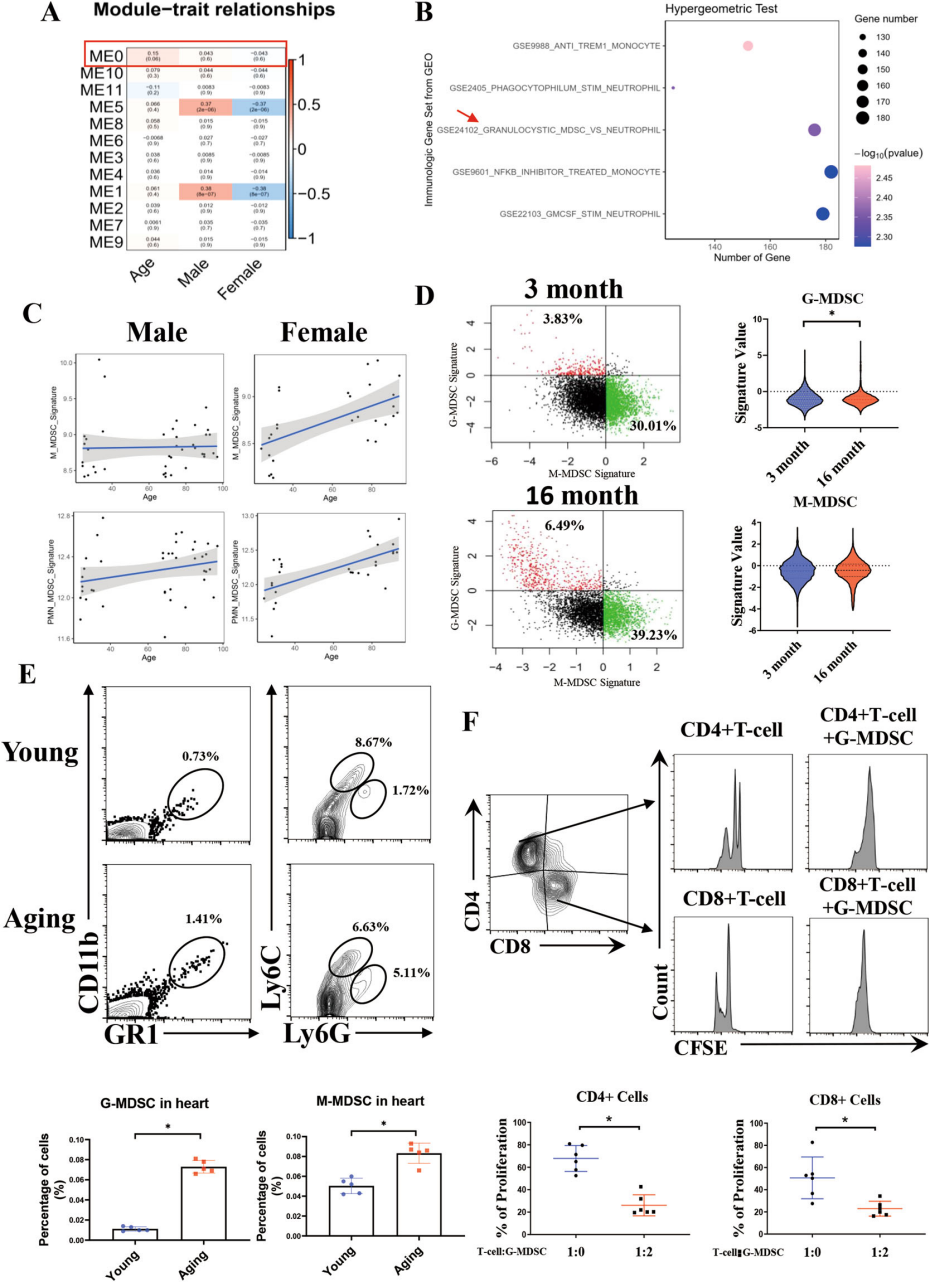
Levels of MDSCs in the heart are positively associated with age. **A** WGCNA of the coexpressed genes from 135 human whole blood transcriptomes from the GEO database (GSE123698). ME0 was related to age (correlation coefficient = 0.15) but not to sex (correlation coefficient = 0.043). Red indicates a positive correlation, and blue indicates a negative correlation. **B** Hypergeometric test of the genes in ME0 based on the immunologic gene set from GSE123698. The size of the dot represents the number of genes. A darker color indicates a lower correlation. **C** The bubble plots show the correlation between the G-MDSC/M-MDSC signature and age in males and females based on GSE123698. **D** The ratio (left) and signature value (right) of the G-MDSCs and M-MDSCs in 3-month-old and 16-month-old mice determined by scRNA-Seq analysis (GSE145477). **E** Representative flow cytometric profile showing the quantitative analysis of Cd11b + Gr1 + Ly6G+ cells and Cd11b + Gr1 + Ly6C+ cells in the hearts of young and aging mice; *n* = 5 per group. **F** CFSE-labeled CD4 + and CD8 + T cells and G-MDSCs separated from hearts were cocultured at a ratio of 2:1 for 24 h. Representative images and quantitative analysis of the proliferation of CD4 + or CD8 + T cells analyzed by flow cytometry; *n* = 6 per group. The data are presented as the means ± SDs. Differences were determined by Student’s *t* test. *P < 0.05.

To explore the dynamic transcriptome of myeloid-derived cells with age, we obtained RNA-Seq data of 135 human whole blood transcriptomes from the GEO database (GSE123698) and performed correlation analysis. The total coexpressed genes were divided into 10 modules by weighted gene coexpression network analysis (WGCNA), and the analysis showed that module 0 (ME0) was closely related to age (correlation coefficient = 0.15) but not to sex (correlation coefficient = 0.043) (Fig. 1A). Then, hypergeometric tests were performed on 913 genes in ME0 according to immunologic gene sets. The bubble plot showed that a series of myeloid-derived cell-related gene sets passed the filtering criteria. Notably, G-MDSC-related genes were significantly linked to ME0 (Fig. 1B). Next, the correlation between the M-MDSC/G-MDSC signature and age was detected by a deconvolution method. Compared with the M-MDSC signature, the G-MDSC signature was associated with age in both male and female individuals, indicating that the correlation between the G-MDSC signature and age was more significant than that of the M-MDSC signature (Fig. 1C). To further verify these findings, through single-cell RNA-Seq (scRNA-Seq) data (GSE145477) of myeloid-derived cells in mice, we calculated the M-MDSC/G-MDSC signature and found that the G-MDSC signature increased in the mice at 16 months compared with that in the mice at 3 months, while the M-MDSC signature remained almost unchanged (Fig. 1D). We therefore examined the changes in G-MDSCs/M-MDSCs in aging mice. Flow cytometric analysis revealed that the level of CD11b + GR1 + Ly6G+ cells (G-MDSCs) was significantly elevated in the hearts of aging mice, while there was no obvious change in CD11b + GR1 + Ly6C+ cells (M-MDSCs) (Fig. 1E), similar results were observed in the spleen, blood, and bone marrow (Supplement Fig. 1). To determine whether these granulocyte-like cells exert an immunosuppressive effect, we cocultured granulocyte-like cells purified from hearts and spleens with CD4 + and CD8 + T cells at a ratio of 2:1. As expected, these granulocyte-like cells strongly inhibited the proliferation of CD4 + T cells and CD8 + T cells (Fig. 1F and Supplement Fig. 2). Overall, our results demonstrated that G-MDSCs are enhanced in human and mouse hearts with aging.

### The G-MDSCs from aging mice induce cardiac fibrosis

**Fig. 2:**
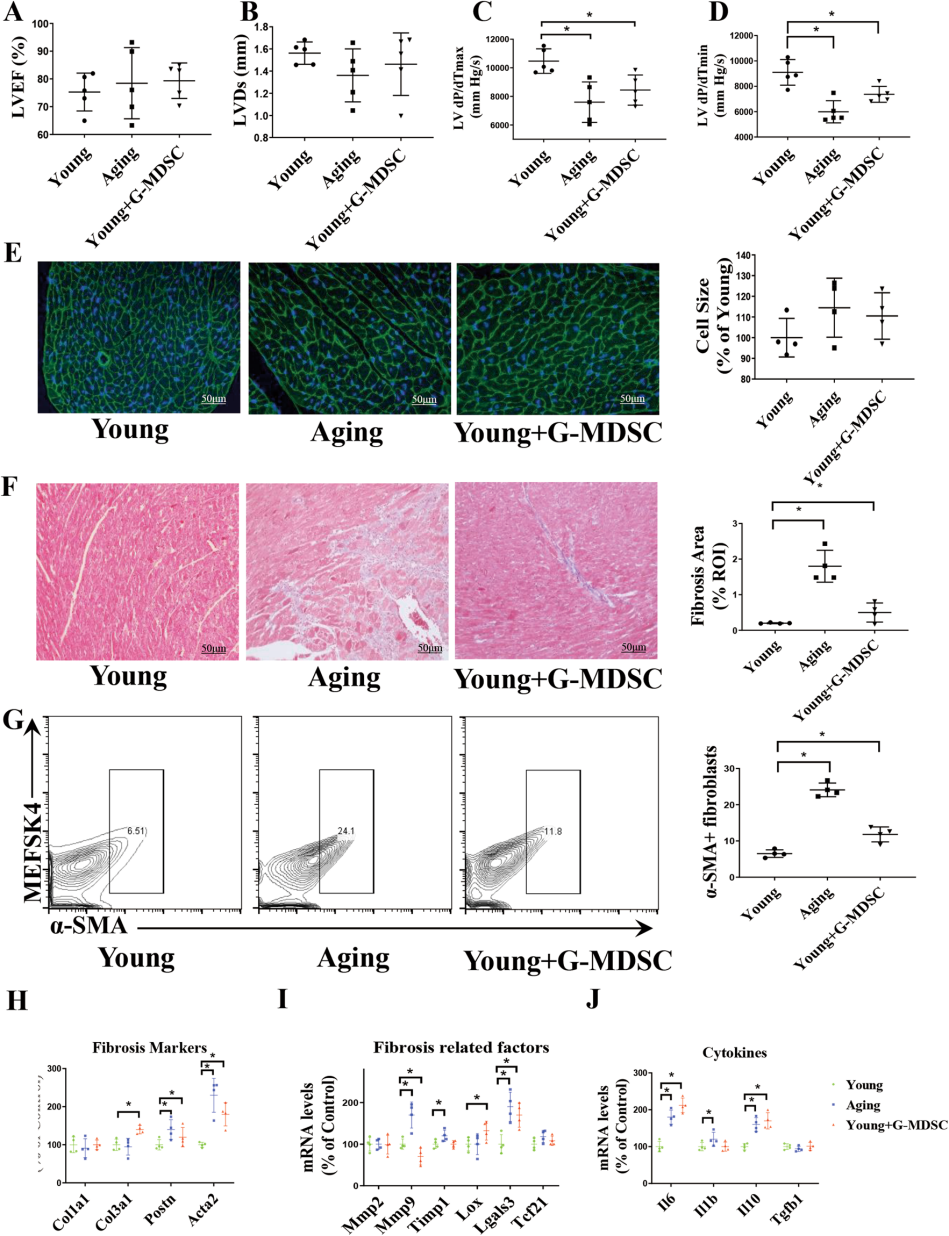
G-MDSCs from aging mice induce cardiac fibrosis. Adoptive transfer of G-MDSCs (1 × 10^7^) was performed in young mice (6 weeks) every 5 days for 40 days. Echocardiography, pathological examination, and qPCR were performed in young mice, old mice (20 months), and young mice that were administered G-MDSCs. **A**–**D** Left ventricular ejection fraction (LVEF; **A**), LV end-systolic diameter (LVDs; **B**), LV dp/dTmax (**C**), and LV dp/dTmin (**D**) of the young mice, aging mice, and young mice with G-MDSCs; *n* = 5 per group. **E** WGA staining revealed the cardiomyocyte cross-sectional area of the young mice, aging mice, and young mice with G-MDSCs. Representative cytograms are shown on the left, and statistical data are shown on the right; *n* = 4 per group. Scale bars, 50 μm. **F** Masson staining revealed fibrotic tissue in the mouse hearts. Representative cytograms are shown on the left, and statistical data are shown on the right; *n* = 4 per group. Scale bars, 50 μm. **G** The ratio of α-SMA + fibroblasts detected by flow cytometry of the young mice, aging mice, and young mice with G-MDSCs. Representative cytograms are shown on the left, and statistical data are shown on the right; *n* = 4 per group. **H**–**J** The mRNA levels of fibrosis markers (Col1a1, Col3a1, Postn, and Acta2; **H**), fibrosis-related factors (Mmp2, Mmp9, Timp1, Lox, Lgals3, and Tcf21; **I**), and cytokines (IL6, IL1b, IL10, and Tgfβ1; **J**) in the hearts of the young mice, aging mice and young mice with G-MDSCs; *n* = 4 per group. The data are presented as the means ± SDs. Differences were determined by one-way ANOVA (for more than 2 groups), and Tukey’s HSD post hoc test was performed. *P < 0.05.

To investigate the effect of the G-MDSCs from aging mice on the heart, we performed adoptive transfer of G-MDSCs (1 × 10^7^) extracted from aging mice (20 months) to young mice (6 weeks) in vivo. We found that the transferred G-MDSCs did not survive for more than 48 h in mice heart (Supplement Fig. 3); thus, the transfer of G-MDSCs was performed through tail vein injection every 5 days for 40 days. Compared with that of the young mice, the diastolic function of the aging mice was substantially reduced, as indicated by obvious decreases in dP/dT max and dP/dT min. Notably, transfer of the G-MDSCs also reduced the diastolic function in the young mice (Fig. 2A–D). Cardiomyocyte hypertrophy and fibrosis are typical pathological features of cardiac diastolic dysfunction. However, WGA staining showed no obvious difference in cardiomyocyte size between the young mice with or without G-MDSCs (Fig. 2E). By Masson staining, we observed a substantial increase in fibrotic areas in the cardiac sections of the aging mice, and similar phenomena were also observed in the mice with transfer of G-MDSCs (Fig. 2F), indicating that G-MDSCs can induce cardiac fibrosis. Through scRNA-Seq data (E-MTAB7869), we observed the enhancement of fibroblasts in aging mouse hearts (Supplement Fig. 4). Hence, we detected the difference in cardiac fibroblast phenotypes between the mice with or without transfer of G-MDSCs. Through flow cytometry, we found that transfer of G-MDSCs significantly increased the number of myofibroblasts (collagen I + α-SMA + ) in the hearts of the young mice (Fig. 2G). Moreover, transfer of G-MDSCs induced strong upregulation of the mRNA expression of fibrotic markers (Col3a1, Postn, Acta2), fibrosis-related factors (MMP9, Lox, and Lgals3), and inflammatory cytokines (IL6 and IL10) (Fig. 2H, I). Interestingly, the change in the Tgf-β level was unremarkable in the aging mice and the young mice with transfer of G-MDSCs (Fig. 2J). Furthermore, we cocultured fibroblasts with granulocyte-like cells from young and aging mice and found that the G-MDSCs from aging mice increased the levels of fibrotic markers (Acta2, Spp1, Fgf2), while the granulocyte-like cells from young mice had no obvious effects (Supplement Fig. 5), revealing that the role of G-MDSCs is due to a functional change and not accumulation. Overall, our in vivo experiments suggested that aging-related G-MDSCs can impair cardiac diastolic function by inducing cardiac fibrosis.

### G-MDSCs induce inflammatory phenotypes and increase the level of OPN in fibroblasts by secreting S100A8/9

**Fig. 3:**
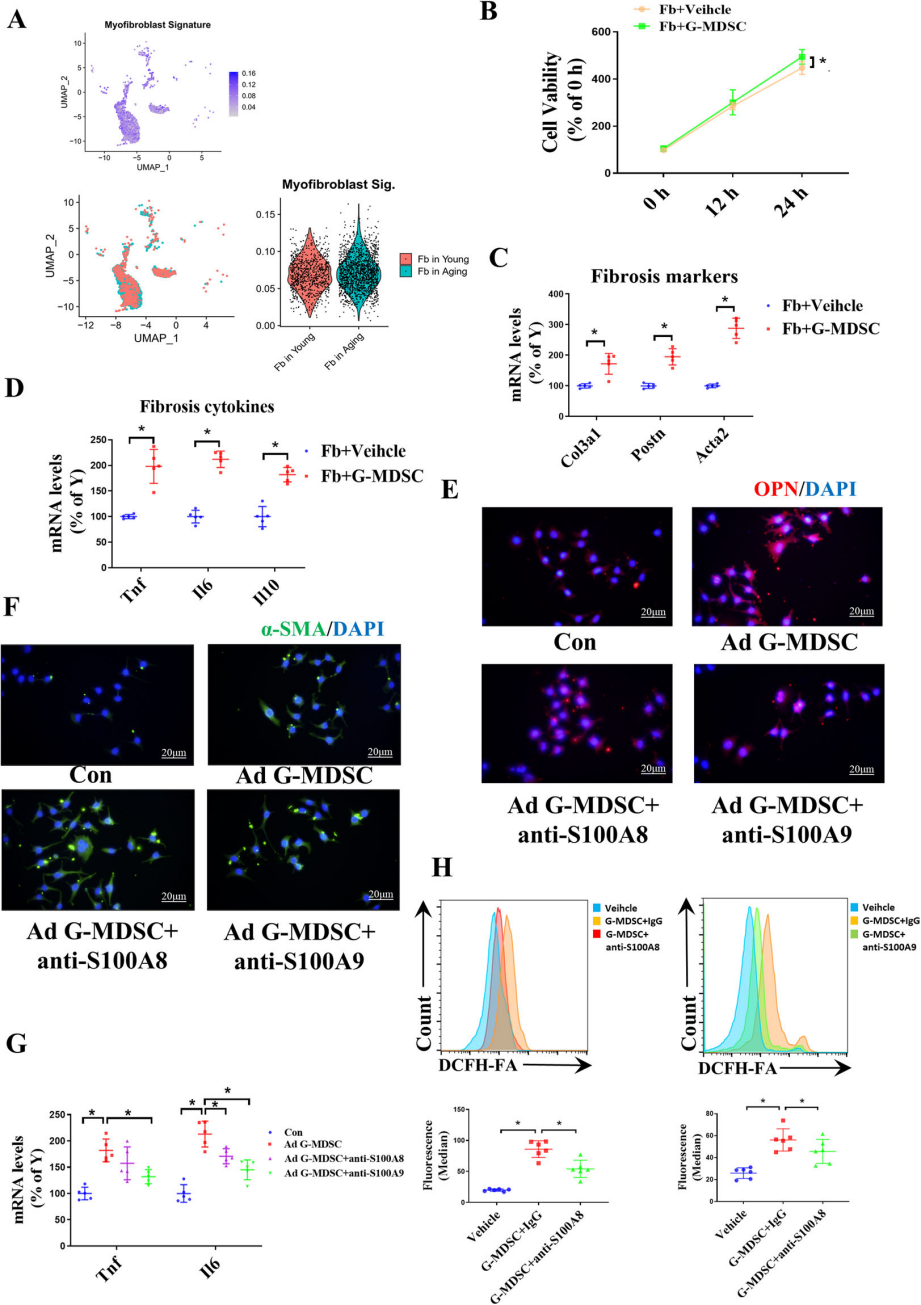
G-MDSCs induce fibroblast inflammatory phenotypes and increase OPN levels. **A** The t-SNE plot showing the significance of myofibroblasts in cardiac fibroblasts of young and aging mice based on the scRNA-seq data set(E-MTAB7869). **B** CCK-8 analysis shows the proliferative potential of the fibroblasts cultured alone or cocultured with G-MDSCs at 12 h and 24 h; *n* = 5 per groups. **C**, **D** The mRNA levels of fibrosis markers (Col3a1, Postn, Acta2; **C**) and inflammatory cytokines (Tnf, IL6, IL10; **D**) in the fibroblasts cultured alone or cocultured with G-MDSCs for 24 h analyzed by qPCR; *n* = 5 per group. **E**–**H** Fibroblasts were cocultured with G-MDSCs for 24 h, and anti-S100A8/anti-S100A9 (50 µg) was added to the culture to neutralize S100A8/A9 secreted by G-MDSCs. Immunofluorescence staining revealed the levels of OPN (**E**) and α-SMA (**F**) in fibroblasts. Scale bars, 20 μm. **G** The mRNA levels of Tnf and IL6 in the fibroblasts of each group were analyzed by qPCR; *n* = 5 per group. **H** Intracellular ROS generation was analyzed by flow cytometry through DCFH-FA staining. Representative cytograms are shown on the top, and statistical data are shown on the bottom; *n* = 6 per group. The data are presented as the means ± SDs. Differences were determined by Student’s *t* test (for 2 groups) or one-way ANOVA (for more than 2 groups), and Tukey’s HSD post hoc test was performed. Repeated measures ANOVA was performed for time series data, and correlations were evaluated by Pearson’s test. *P < 0.05.

To help elucidate the mechanism of aging-related G-MDSC-mediated cardiac fibrosis, we explored the phenotypes of cardiac fibroblasts in aging mice from the scRNA-Seq database (E-MTAB7869). Through cell type recognition, we found that the proportion of fibroblasts significantly increased in aging hearts compared with young hearts (Fig. 3A). Fibrotic markers (Acta2, Fn1, and Col1a1) were significantly increased in the aging hearts (Supplement Fig. 6). G-MDSCs promoted the proliferation of fibroblasts (Fig. 3B) and enhanced the levels of fibrotic markers (Col3a1, Postn, and Acta2) (Fig. 3C). Interestingly, we found that G-MDSCs increased the levels of cytokines, including IL10, Tnf, and IL6 (Fig. 3D). Previous studies have reported that OPN is required for fibroblast activation and promotes aging-related cardiac fibrosis^13^. We therefore tested the levels of OPN and α-SMA in fibroblasts. By immunofluorescence staining, we observed that the levels of α-SMA and OPN were increased in the fibroblasts cocultured with G-MDSCs (Fig. 3E, F). These results demonstrated that G-MDSCs could promote cardiac fibrosis. S100A8/A9, biomarkers of MDSCs, were reported to upregulated proinflammatory genes in the inflammatory response^14^. The single-cell RNA-seq (GSE145477) analysis suggested that S100A8/9 expression levels were higher in G-MDSCs (Supplement Fig. 7A) than that in M-MDSCs (Supplement Fig. 7B), which is consistent with our qPCR results (Supplement Fig. 7C) and the study by Veglia, F et al.^10^. In addition, we also found that the level of S100A8/9 in granulocyte-like cells (CD11b + Gr1 + Ly6G + ) was higher in aging mice than that in young mice (Supplement Fig. 8A, B). Compared with S100A8, the level of S100A9 increased more significantly in granulocyte-like cells (Supplement Fig. 8C).

Neutralizing S100A8 and S100A9 decreased the levels of proinflammatory cytokines (Tnf and IL6) (Fig. 3G). By flow cytometry, we found that G-MDSCs increased reactive oxygen species (ROS) generation and that neutralizing S100A8/A9 reduced the level of intracellular ROS (Fig. 3H). Interestingly, immunofluorescence revealed that neutralizing S100A8 and S100A9 decreased the OPN level in fibroblasts but had no obvious effect on the level of α-SMA (Fig. 3E, F). Altogether, our results indicated that G-MDSCs can promote inflammatory phenotypes and increase the level of OPN in fibroblasts by releasing S100A8/A9.

### Aging-related G-MDSCs inhibit the cellular senescence and apoptosis of fibroblasts

**Fig. 4:**
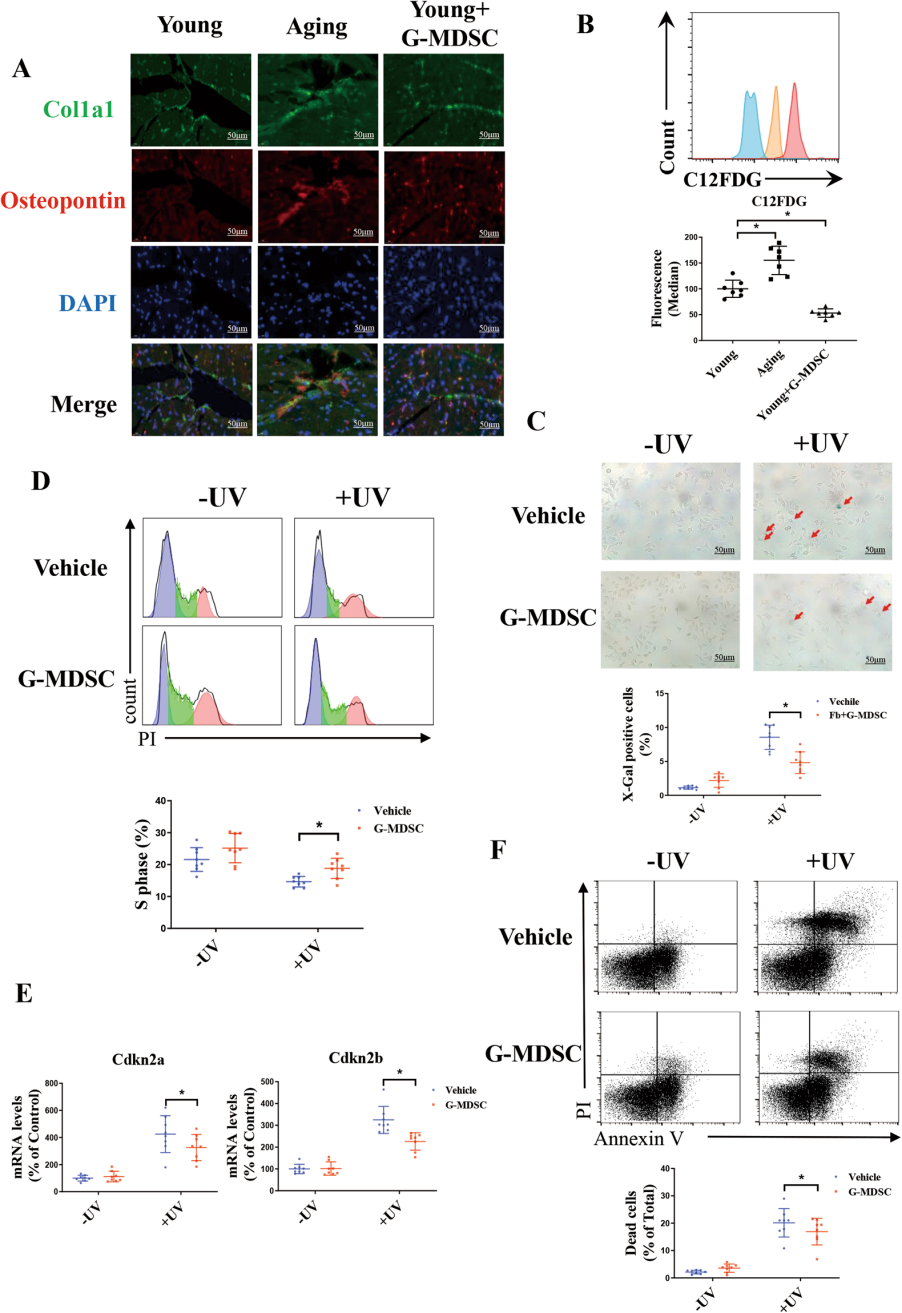
Aging-related G-MDSCs suppress fibroblast senescence and apoptosis. **A**, **B** Young fibroblasts were cultured with aging-related G-MDSCs for 24 h to determine the changes induced by aging-related G-MDSCs. **A** Immunofluorescence results revealed the level of OPN in fibroblasts. Scale bars, 50 μm. **B** The levels of β-galactosidase in fibroblasts analyzed by flow cytometry through C12FDG staining. Representative cytograms are shown on the top, and statistical data are shown on the bottom; *n* = 7 per group. **C**–**F** The senescent fibroblast model was induced by UV treatment. Then, we investigated the influence of G-MDSCs on fibroblast senescence and apoptosis. **C** Representative images of senescent fibroblasts detected by X-gal staining are shown on the top, and statistical data are shown on the bottom. Red arrows represent X-Gal^+^ fibroblasts; *n* = 8 per group. Scale bars, 50 μm. **D** Cell cycle distribution and the percentage of fibroblasts in S phase were detected by flow cytometry. Representative cytograms are shown on the top, and statistical data are shown on the bottom; *n* = 8 per group. **E** The mRNA levels of the senescence markers Cdkn2a and Cdkn2b in the fibroblasts with or without UV treatment analyzed by qPCR; *n* = 8 per group. **F** The percentage of apoptotic fibroblasts was detected by flow cytometry using Annexin V-FITC and PI staining. Representative cytograms are shown on the top, and statistical data are shown on the bottom; *n* = 8 per group. The data are presented as the means ± SDs. Differences were determined by an unpaired *t* test, one-way ANOVA (more than 2 groups) or two-way ANOVA (more than 2 factors), and Tukey’s HSD post hoc test (for one-way ANOVA) or a Sidak HSD post hoc test (for two-way ANOVA) was performed. *P < 0.05.

Through scRNA-Seq data (E-MTAB7869), we found that the levels of inflammatory cytokines (IL6, IL1b, IL10, and Tnf) and senescence markers (Ccl2, Cdkn2a, Cdkn2b, and Mki67) increased in the cardiac fibroblasts of aging mice, confirming cell senescence (Supplement Fig. 9). Immunofluorescence staining indicated that the level of OPN, a marker of cell senescence that is involved in fibrosis, increased in the fibroblasts from young mice cultured with G-MDSCs (Fig. 4A). Therefore, further explorations were conducted on the effect of G-MDSCs on fibroblast senescence. Interestingly, G-MDSCs decreased the level of β-galactosidase in fibroblasts (Fig. 4B), suggesting that G-MDSCs may blunt fibroblast senescence. We next induced cellular senescence of fibroblasts by ultraviolet (UV) treatment in vitro. The β-galactosidase staining results showed that G-MDSCs decreased fibroblast senescence induced by UV treatment (Fig. 4C). As demonstrated by cell cycle assays, the fibroblasts cocultured with G-MDSCs showed significant cell cycle arrest at S phase compared with the fibroblasts cultured alone, regardless of UV treatment (Fig. 4D). Similarly, G-MDSCs suppressed UV-induced mRNA upregulation of the senescence markers Cdkn2a and Cdkn2b. (Fig. 4E). Furthermore, flow cytometry revealed that G-MDSCs inhibited fibroblast apoptosis induced by UV treatment (Fig. 4F). Overall, our data indicated that aging-related G-MDSCs could inhibit fibroblast senescence and apoptosis by inducing cell cycle arrest.

### G-MDSCs have antisenescence and antiapoptotic effects on fibroblasts by upregulating FGF2 levels in fibroblasts

**Fig. 5:**
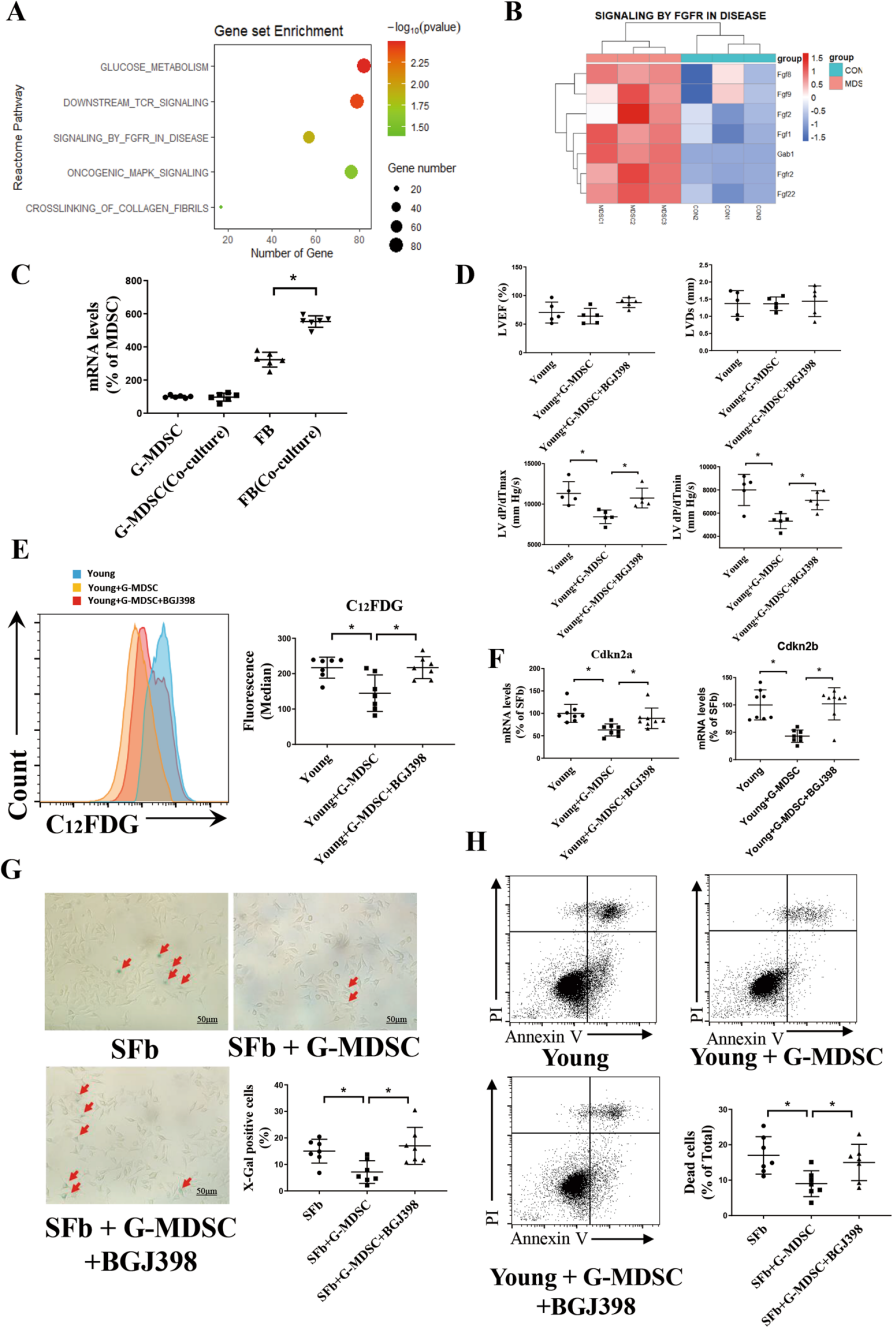
FGF2 secreted by fibroblasts is involved in G-MDSC-mediated suppression of senescence and apoptosis. **A** KEGG pathway enrichment showing signaling pathways related to G-MDSCs. **B** Heatmap showing the expression levels of genes involved in signaling by the FGFR pathway in the MDSC-treated mice and the control mice. Red indicates a positive correlation, and blue indicates a negative correlation. **C** The mRNA levels of FGF2 in the fibroblasts and G-MDSCs cultured alone or cocultured with each other for 24 h analyzed by qPCR; *n* = 6 per group. **D** LVEF, LVDs, LV dp/dTmax, and LV dp/dTmin of the mice administered G-MDSCs with or without BGJ398 (5 µm); *n* = 5 per group. **E**–**H** Senescent fibroblasts were cocultured with G-MDSCs for 24 h, and BGJ398 (5 µm) was used to neutralize FGF2. **E** The levels of β-galactosidase in fibroblasts analyzed by flow cytometry through C12FDG staining. Representative cytograms are shown on the left, and statistical data are shown on the right; *n* = 7 per group. **F** The mRNA levels of the senescence markers Cdkn2a and Cdkn2b in fibroblasts; *n* = 8 per group. **G** Representative images and statistical data of senescent fibroblasts using X-gal staining. Red arrows represent X-Gal^+^ fibroblasts; *n* = 7 per group. Scale bars, 50 μm. **H** Representative flow cytometric profile and statistical data of fibroblast apoptosis through Annexin V-FITC and PI staining; *n* = 7 per group. The data are presented as the means ± SDs. The data are presented as the means ± SDs. Differences were determined by one-way ANOVA (more than 2 groups), and Tukey’s HSD post hoc test was performed. *P < 0.05.

We used RNA-Seq to investigate the mechanism underlying the effect of G-MDSCs on cardiac fibroblasts. GSEA indicated that several signaling pathways were enhanced in the fibroblasts cocultured with G-MDSCs. Among these pathways, FGFR signaling is believed to be involved in fibrosis (Fig. 5A). As shown in the heatmap, the levels of FGF family members strongly increased in the mice treated with G-MDSCs (Fig. 5B). Among these factors, FGF2 was selected because of its enrichment in cardiac tissue. To explore the origin of FGF2, we examined the mRNA levels of FGF2 in fibroblasts and G-MDSCs. Interestingly, the level of FGF2 was lower in G-MDSCs and higher in fibroblasts, especially when they were cocultured with G-MDSCs. (Fig. 5C). Our data revealed that G-MDSCs can upregulate the level of FGF2 in fibroblasts. Next, we performed RNA-Seq on fibroblasts cocultured with G-MDSCs or 100 μg/mL S100A8/9 for 48 h to explore whether S100A8/9 affects the FGF2 level. The heatmap indicates that the S100A8/9 treatment significantly increased the levels of Spp1 (OPN) of fibroblasts but downregulated Fgf2 expression (Supplement Fig. 10A), which is consistent with our qPCR results (Supplement Fig. 10B). Thus, the above results suggested that the level of FGF2 in fibroblasts was independent of S100A8/9. To further investigate whether FGF2 inhibits fibroblast senescence and apoptosis, we performed adoptive transfer of G-MDSCs with/without BGJ398 (FGF2 inhibitor) treatment in young mice. Echocardiography revealed that neutralization of FGF2 partly alleviated the diastolic dysfunction induced by the transfer of G-MDSCs, as indicated by the increased dP/dT max and dP/dT min values (Fig. 5D). Flow cytometric analysis of β-galactosidase showed that neutralization of FGF2 partly relieved the G-MDSC-mediated antisenescence effects (Fig. 5E). Neutralization of FGF2 increased the mRNA levels of the senescence markers Cdkn2a and Cdkn2b (Fig. 5F) but had little effect on the mRNA levels of inflammatory cytokines (Supplementary Fig. 11). Then, we examined the senescence and apoptotic phenotypes of fibroblasts in vitro. β-Galactosidase staining of senescent fibroblasts showed that neutralization of FGF2 induced an increase in senescent cells (Fig. 5G). Similarly, flow cytometry showed that neutralization of FGF2 increased the ratio of apoptotic fibroblasts (Fig. 5H). Thus, our data revealed that G-MDSCs mediate antisenescence and antiapoptotic functions by enhancing the release of FGF2 in fibroblasts.

### SOX9 is required for FGF2-mediated antisenescence and antiapoptotic functions

**Fig. 6:**
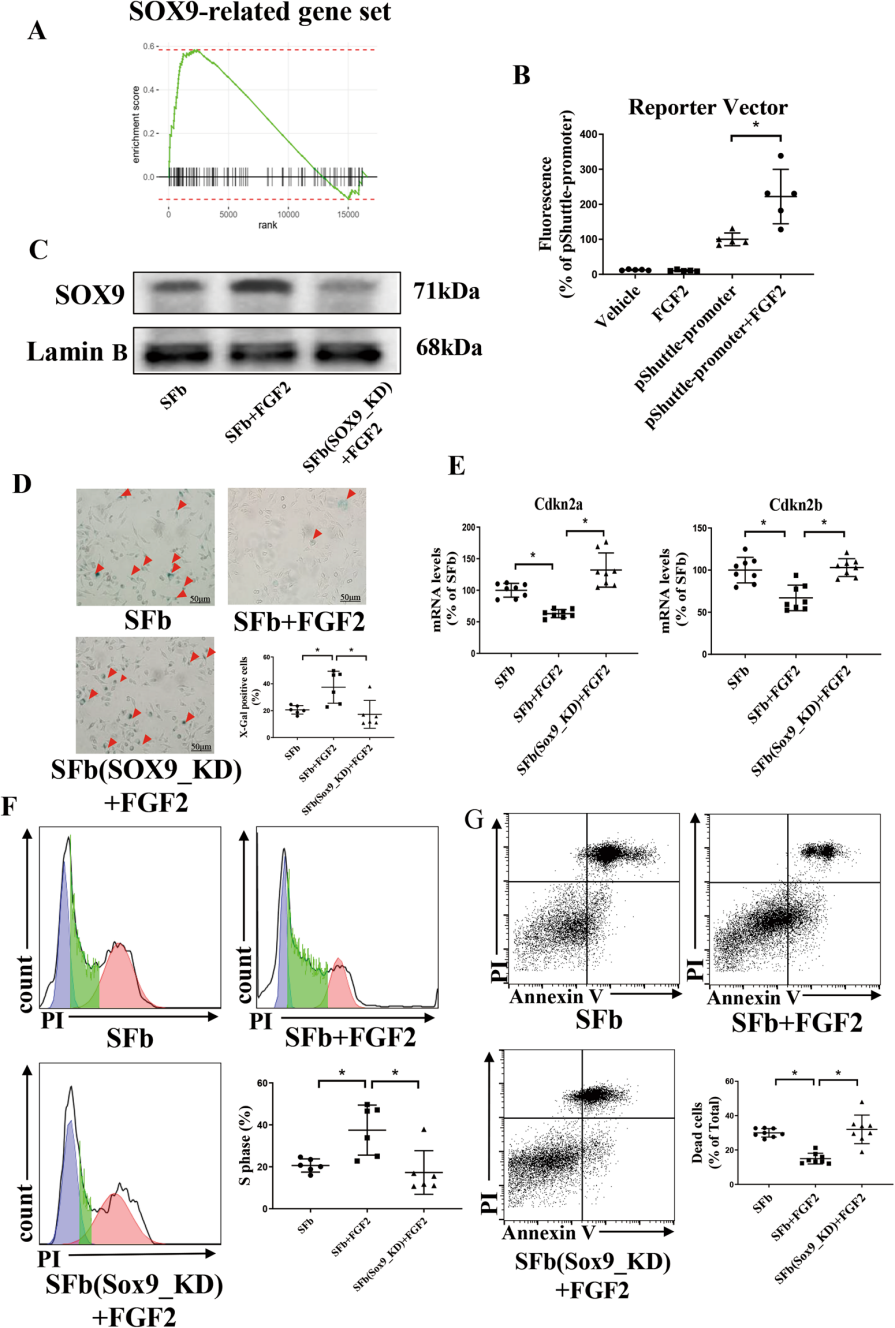
The profibrotic factor SOX9, induced by FGF2 elevation, is required for G-MDSC-mediated suppression of senescence and apoptosis. **A** GSEA showing the SOX9-related gene set enriched in the fibroblasts treated with FGF2. **B** Luciferase activity driven by the SOX9 motif in the fibroblasts treated with FGF2. An asterisk indicates a significant difference vs. the pShuttle promoter; *n* = 5 per group. **C**–**G** The fibroblasts with SOX9 knockdown in vitro were cultured with medium supplemented with FGF2 (4 ng/mL) for 24 h. **C** Western blotting analysis showing the expression level of SOX9 in senescent fibroblasts. **D** Representative X-gal staining images (top) and statistical data (bottom) on fibroblast senescence; *n* = 6 per group. Scale bars, 50 μm. **E** The mRNA levels of the senescence markers Cdkn2a and Cdkn2b in senescent fibroblasts; *n* = 8 per group. **F** Cell cycle distribution and the percentage of fibroblasts in S phase were detected by flow cytometry through PI staining; *n* = 6 per group. **G** Representative flow cytometric images and statistical data of apoptosis rates of senescent fibroblasts determined by Annexin V-FITC and PI staining; *n* = 8 per group. The data are presented as the means ± SDs. Differences were determined by one-way ANOVA (more than 2 groups), and Tukey’s HSD post hoc test was performed. *P < 0.05.

To explore the downstream targets of FGF2, we performed further experiments. GSEA of transcriptome data showed that SOX9, which has a profibrotic role, was enriched in the mice administered G-MDSCs (Fig. 6A). To investigate whether FGF2-induced fibrosis was dependent on SOX9, we transfected fibroblasts with a luciferase reporter plasmid (containing 12 repeats of a 48-bp COL2A1 intron 1 enhancer element, which is known to be activated by SOX9). As expected, FGF2 increased the luciferase activity driven by SOX9 (Fig. 6B). Western blotting results also showed that FGF2 treatment increased the level of SOX9 in fibroblast nuclei (Fig. 6C). The RNA-seq analysis showed that the Sox9-related gene set was significantly enriched with the G-MDSC treatment, but showed no significance between the S100A8/9 treatment and control group (Supplement Fig. 10C), suggesting that SOX9 expression was independent of S100A8/9. Then, we examined the senescence and apoptotic phenotypes of fibroblasts in vitro to verify the link between FGF2 and SOX9. β-Galactosidase staining showed that SOX9 knockdown enhanced the number of senescent cells (Fig. 6D). SOX9 knockdown increased the mRNA levels of the senescence markers Cdkn2a and Cdkn2b (Fig. 6E). As shown by the cell cycle assay, SOX9 knockdown alleviated the arrest at S phase induced by FGF2 (Fig. 6F). Moreover, SOX9 knockdown increased the number of apoptotic fibroblasts compared with those of the fibroblasts cultured with FGF2 alone (Fig. 6G). In conclusion, our data revealed that SOX9 is a downstream gene of FGF2.

### SOX9 is required for G-MDSC-mediated cardiac fibrosis

**Fig. 7:**
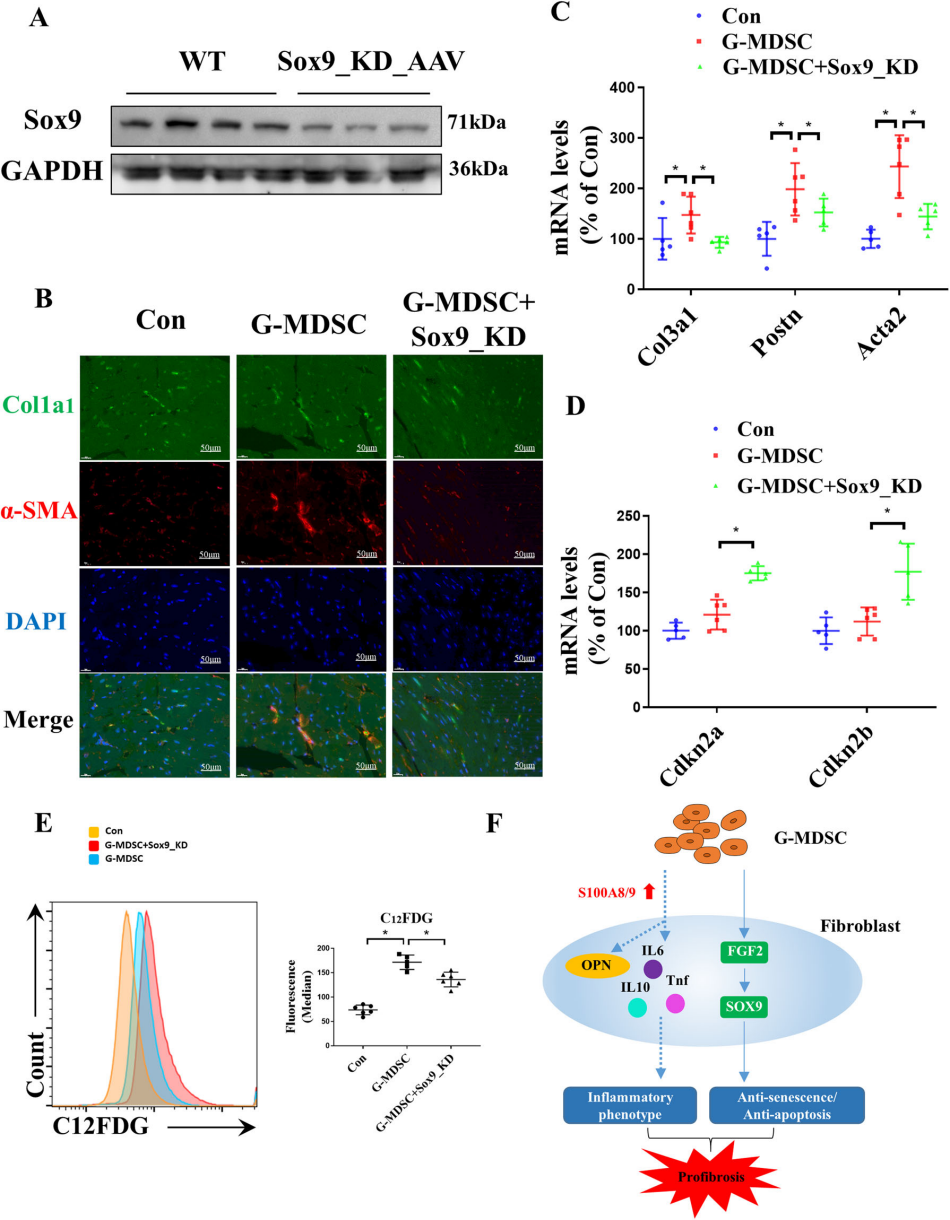
SOX9 knockdown alleviates the fibrotic phenotypes induced by G-MDSCs in vivo. Induction of SOX9 knockdown in MDSC-treated mice through injection of AAV harboring the SOX9-KD plasmid. **A** Western blotting analysis showing the expression levels of SOX9 in the WT and SOX9_KD hearts. **B** Representative immunofluorescence images of the level of α-SMA in the control, G-MDSC, and SOX9_KD hearts. Scale bars, 50 μm. **C** The mRNA levels of fibrosis markers (Col3a1, Postn, and Acta2) in mouse hearts analyzed by qPCR; *n* = 5–6 per group. **D** The mRNA levels of the senescence markers Cdkn2a and Cdkn2b in mouse hearts analyzed by qPCR; *n* = 5–6 per group. **E** The levels of β-galactosidase in fibroblasts analyzed by flow cytometry through C12FDG staining. Representative cytograms are shown on the left, and statistical data are shown on the right; *n* = 5–6 per group. **F** Diagram of the mechanism by which G-MDSCs act on fibroblasts. The data are presented as the means ± SDs. Differences were determined by an unpaired *t*-test or two-way ANOVA (more than 2 factors), and a Sidak HSD post hoc test was performed. *P < 0.05.

Next, we knocked down SOX9 expression in the G-MDSC-transferred mouse hearts by injecting adeno-associated virus (AAV) harboring the SOX9-KD plasmid in the mice, and the results were confirmed by Western blot analysis (Fig. 7A). Then, we analyzed the effect of SOX9 knockdown on cardiac fibrosis. Immunofluorescence analysis revealed that the level of α-SMA in the SOX9_KD hearts returned to a level similar to that of the control group, indicating that fibroblast activation mediated by G-MDSCs was suppressed (Fig. 7B). Similarly, the mRNA levels of fibrosis markers (Col3a1, Postn, Acta2) decreased in the SOX9_KD mouse hearts (Fig. 7C). Moreover, SOX9 knockdown induced elevated levels of Cdkn2a, Cdkn2b (Fig. 7D), and β-galactosidase (Fig. 7E) in fibroblasts, indicating that SOX9 is involved in G-MDSC-mediated suppression of cellular senescence. These results demonstrated that SOX9 is required for the G-MDSC-mediated profibrotic functions.

## Discussion

In our study, we revealed the role of G-MDSCs as an important profibrotic factor in the aging heart. We found that G-MDSCs can promote fibroblast proliferation and increase the levels of fibrosis markers. S100A8 and S100A9 released by G-MDSCs mediated the inflammatory phenotypes and increased the OPN level in fibroblasts. In addition, we demonstrated that G-MDSCs can suppress fibroblast senescence and apoptosis via FGF2-SOX9 signaling. Since aging-related fibrosis is an important pathological factor of HFpEF, G-MDSCs could be a new antifibrogenic therapeutic target for HFpEF.

MDSCs develop from common myeloid progenitor cells during the myelopoietic process and possess a powerful array of immune-suppressive mechanisms^15^. These cells are recruited to inflammatory tissue to suppress acute inflammation by inhibiting the functions of innate and adaptive immunity. Accumulating evidence has indicated that the numbers of MDSCs increase in the spleen, bone marrow, peripheral lymph nodes, and peripheral blood with aging^16–20^. In our study, we confirmed that G-MDSCs, rather than M-MDSCs, accumulated in aging hearts and verified their immunosuppressive function through their suppressive effect on T-cell proliferation. The accumulation of G-MDSCs in aging hearts may be associated with aging-associated chronic low-grade inflammation, which is always accompanied by aging progression^21^. Low levels of persistent inflammation have been verified to be related to pathophysiological cardiovascular diseases (CVDs), such as hypertension and arteriosclerosis^22,23^. In detail, the secretome of the senescence-associated secretory phenotype (SASP) and the high levels of proinflammatory cytokines in circulation, the most typical inducers of MDSCs, can activate the proliferation and recruitment of MDSCs in inflammatory tissue^24,25^. As G-MDSCs accumulate in aging hearts, their role in senile CVDs remains unclear. By transferring G-MDSCs, we found that these cells can substantially decrease cardiac diastolic function by inducing cardiac fibrosis, which is similar to the pathological changes in aging hearts. As relevant studies have shown that MDSCs are closely related to pulmonary fibrosis^26^, we next explored the relationship between G-MDSCs and fibroblast phenotypes. As expected, we demonstrated that G-MDSCs can activate the proliferation of fibroblasts. In addition, G-MDSCs increased the levels of the fibrosis markers Col3a1, Postn, and Acta2 in fibroblasts, indicating that G-MDSCs play a crucial role in cardiac fibrosis.

S100A8 and S100A9, also known as migration inhibitory factor-related proteins 8 (MRP8) and 14 (MRP14), are biomarkers of MDSCs^10^. Extracellular S100A8/A9 promote the production of inflammatory cytokines by binding to two pattern recognition receptors: Toll-like receptor 4 (TLR4) and receptor of advanced glycation endproducts (RAGE)^27–29^. Notably, S100A8/A9 can induce the accumulation and migration of MDSCs through multiple mechanisms^30,31^. Here, our data revealed that release of S100A8/A9 by G-MDSCs promoted the secretion of proinflammatory cytokines and elevated OPN levels in fibroblasts. Accumulation of MDSCs in aging tissue is believed to prevent excessive inflammation caused by aging^15^. In contrast, recent studies have shown that MDSCs can aggravate chronic inflammation by inhibiting both innate and adaptive immunity in chronic inflammatory tissue or aging tissue^32–35^. We observed that G-MDSCs increased the levels of proinflammatory cytokines, such as IL-6, IL-10, and TNF-α in fibroblasts. The aging process was shown to be associated with a balance between proinflammatory and anti-inflammatory responses, and MDSCs play an important role in this process^21,36,37^. A previous study indicated that activated MDSCs suppress the functions of invading inflammatory cells, limiting the excessive inflammatory response but promoting the presence of low-grade chronic inflammation^25^. Thus, our results suggested that G-MDSCs promote the inflammatory phenotypes of fibroblasts, indicating that the role of G-MDSCs in the immune microenvironment of aging tissue deserves further exploration. OPN is a proinflammatory cytokine and a crucial matricellular protein of the extracellular matrix (ECM). As documented by several studies, OPN is essential for fibroblast activation^38–40^. Daigo Sawaki et al. confirmed that the accumulation of OPN, derived from visceral adipose tissue, in aging hearts can promote cardiac fibrosis^13^, identifying the origin of OPN. In addition, increased cardiac stiffness induced by a high level of OPN represents an increased risk of cardiac diastolic dysfunction, which is an important pathological characteristic of HFpEF^41,42^. In summary, our data suggested that extracellular S100A8 and S100A9 are involved in G-MDSC-mediated profibrotic progression.

Another interesting finding is that G-MDSCs trigger fibroblast cell cycle self-renewal and antiapoptotic effects in aging hearts. With increasing age, fibroblasts also exhibit aging phenotypes, such as galactosidase enhancement and cell cycle arrest. Our results confirmed that G-MDSCs can suppress fibroblast senescence and apoptosis. According to current studies, fibroblast senescence is beneficial in attenuating cardiac fibrosis^13^, suggesting that the induction of fibroblast senescence may be a strategy to inhibit tissue fibrosis. Recent studies have revealed that TGF-β expression was downregulated in fibrotic tissue of aging mice, which is consistent with our results. Therefore, further elucidation of the mechanism of the G-MDSC-mediated profibrotic function is needed. Our data identified a novel FGF2-SOX9 signaling axis in fibroblasts (Fig. 7F). FGF2 is an endogenous heparin-binding multifunctional growth factor expressed and secreted predominantly by cardiac nonmyocytes (fibroblasts) in the heart and has long been known to stimulate the proliferation of fibroblasts^43^. SOX9 plays an essential role during mammalian development, in which it crucially regulates chondrogenesis and sex differentiation^44,45^. SOX9 was shown to be an important transcription factor in cardiac fibrosis and is mainly active in fibroblasts^46^. Through regulation by G-MDSCs, FGF2 secretion is increased in fibroblasts, where it upregulates the SOX9 gene, resulting in cell cycle self-renewal and antiapoptotic effects in fibroblasts. However, the mechanism by which G-MDSCs act on fibroblasts to release FGF2 remains unclear and deserves further exploration. In summary, our data revealed that G-MDSCs can suppress fibroblast senescence and apoptosis by regulating fibroblast FGF2-SOX9 signaling.

Notably, Zhou, L. et al. have reported that MDSCs (CD11b + Gr1 + ) show a cardioprotective effect in heart failure models through their antihypertrophic and anti-inflammatory effects^47^. The differences between the two studies may be explained by the following points. First, Zhou, L. et al. performed the experiments on mice with acute or chronic pressure overload-induced heart failure, which was characterized by excessive activation of the inflammation process^48^. Due to their immunosuppression, MDSCs may alleviate cardiac injury by reducing the levels of pro-inflammatory cytokines. Second, Zhou, L. et al. found that MDSCs showed an antihypertrophic effect on cardiomyocytes. However, our study found that G-MDSC aggravated stiffness of cardiac fibroblasts, which is a salient feature of age-related cardiac dysfunction^35^. Third, Zhou, L. et al. suggested that MDSC exerted cardioprotective effects through nitric oxide, which is synthesized more in M-MDSCs than in G-MDSCs^10^. Taken together, these discrepancies may partly explain the differences between the two studies.

Although our data suggested that the transferred G-MDSCs in heart cannot survive for more than 48 h, we still cannot completely rule out the effect of excessive cell accumulation. Nevertheless, our results provide new insight into the pathology of HFpEF and identify the role of G-MDSCs in inducing aging-related cardiac fibrosis through release of S100A8/A9 and regulation of FGF2-SOX9 signaling.

## Methods

### Cell isolation and culture

Murine MDSCs were isolated from the spleens of aging C57BL/6 mice (20 months). The spleens were filtered by a cell strainer with 40 μm nylon. Then, the red blood cells were eliminated by red blood cell lysis. G-MDSCs were defined as CD11b + Ly6Clow cells and obtained using fluorescence-activated cell sorting; this method provides nearly 90% CD11b + Gr1 + Ly6G+ cell purity assessed by flow cytometry. The immunosuppression of G-MDSCs was detected by a T-cell proliferation assay.

Mouse fibroblasts were isolated from 5-week-old C57BL/6 mice using enzymatic digestion according to our previous protocol^49^. Mouse heart pieces were enzymatically digested with stirring for 10 min, and the supernatant was collected. The different cell types were isolated by Percoll gradient separation (top Percoll layer: 55%; bottom Percoll layer: 65%). We collected the intermediate layer of cardiomyocytes and the upper layer of cardiac fibroblasts.

The cells were cultured in medium containing 10% fetal bovine serum and 1% streptomycin/penicillin and incubated at 37 °C with 5% CO_2_.

### Animal experiments and adoptive transfer

Young (6 weeks) and aging (20 months) male C57BL/6 mice [obtained from the Experimental Animal Center of Guangzhou University of Chinese Medicine, China, certification no. SCXK (Yue) 2016-0168] weighing 20 ± 1 g were housed in an individual ventilated cage system. The mice were maintained according to the Guidelines for the Care and Use of Laboratory Animals formulated by the Ministry of Science and Technology of China, and all experimental procedures were approved by the Ethics Committee of Guangzhou University of Chinese Medicine. All animals were housed at an ambient temperature of 21 °C under a 12/12 h light–dark schedule and maintained on food formulated according to the American Institute of Nutrition for Rodent Diets, with ad libitum access to water. Randomization was used to assign samples to the experimental groups for all in vivo studies.

For adoptive transfer, G-MDSCs (CD11b + Ly6Clow) were isolated from the spleens of aging C57BL/6 mice (20 months), this method provides nearly 90% CD11b + Gr1 + Ly6G+ cell purity assessed by flow cytometry. G-MDSCs (1 × 10^7^) were injected through the tail vein of recipient mice (more than 5 young mice per group; randomly assigned, not a blinded method) every 5 days during the experiments. At the end of the experiments, the recipient mice were euthanized.

### RNA-Seq and analysis

Total mRNA was extracted from the mouse hearts using an RNA extraction kit (Qiagen K.K., Tokyo, Japan). RNA-Seq was performed using an Ion Proton system for next-generation sequencing, according to the manufacturer’s instructions. Sequenced reads were mapped to the mm9 genome using the Ion Torrent TMAP aligner with the ‘map4’ option. The RNA-Seq reads that were aligned against the exon regions of genes were quantified with HTSeq-Count in the RefSeq mm9 annotation. Gene set enrichment analysis (GSEA) was performed on mRNA expression datasets using our gene set data. Gene signatures were considered enriched if the false discovery rate (FDR) *q*-values and the familywise error rate (FWER) *p*-values showed significant differences.

### Flow cytometric analysis

For analysis of immunomarkers, the cells were suspended in PBS with 0.5% serum and stained with fluorescent antibodies (eBioscience, Inc., San Diego, USA) against CD11b (Cat: 25-0118-42), Gr1 (Cat: 48-5931-82), Ly6C (Cat: 12-5932-82), Ly6G (Cat: 11-9668-82), CD4 (Cat: 11-0041-82), and CD8 (Cat: 48-0081-82). For analysis of fibrosis markers, cardiac cells were isolated as previously described. The cells were stained with primary antibodies (Affinity, Inc., USA) against collagen I (Cat: AF0134) and α-SMA (Cat: BF9212) and secondary fluorescent antibodies. Flow cytometric analysis or sorting was performed by a flow cytometer (MoFlo Astrios EQ, Beckman Coulter, Indianapolis, IN, USA). All the flow cytometric results were analyzed with Summit Software Version 5.2 (Beckman).

### Beta-galactosidase activity assay

Cells cocultured in vitro were fixed with 0.5% paraformaldehyde and stained with 1 mg/mL 5-bromo-4-chloro-3-indoyl β-D-galactopyranoside (X-gal) for 24 h. The stained cells (blue) were imaged and quantified by differential interference contrast brightfield microscopy.

For the beta-galactosidase activity of cardiac fibroblasts in vivo, 5-dodecanoylaminofluorescein di-β-D-galactopyranoside (C_12_FDG) was used according to a previous protocol^49^. Briefly, cardiac fibroblasts were isolated using enzymatic digestion and Percoll gradient separation. Then, the cells were treatment with 50 μM C_12_FDG for 2 h. Flow cytometric analysis was used to estimate the relative β-galactosidase activity based on the green fluorescence intensity.

### Echocardiography

Echocardiography was performed using a Vevo 2100 Imaging System (VisualSonics, Inc., Toronto, Canada). All parameters were automatically obtained using the LV Trace measurement tool in the Cardiac Package and the Short Axis (SAX) module in Vevo 2100 analysis software. The measurements were performed using a single-blinded method.

### Western blotting

The protein content was quantified by the BCA method, and 100 μg per sample was loaded onto 12% SDS polyacrylamide gels. After electrophoresis, the proteins were transferred to polyvinylidene fluoride (PVDF) membranes, which were blocked with 5% BSA and probed with primary antibodies (Affinity, 1:1000, SOX9: Cat: AF6330; Lamin-B: Cat: DF7356). After the PVDF membranes were washed, they were incubated with a secondary antibody (Affinity, 1:2000, Cat. No. S0001) for 2 h. Finally, the blots on the membranes were scanned with a charge-coupled device (CCD) system (ImageStation 2000 MM, Kodak, USA).

### Real-time fluorescence quantitative PCR

The TRIzol method was used to extract total RNA from cells (Life Technologies, USA). A cDNA Synthesis Kit (TaKaRa, Japan) was used to synthesize cDNA for real-time fluorescence quantitative PCR (qPCR) assays. PCR amplification was carried out on a thermal cycler in a reaction volume of 20 µL containing SYBR Green (TaKaRa, Japan) using a MyiQ real-time PCR detection system (Bio-Rad). The data were analyzed using the 2^-ΔΔCt^ method, and the gene primers are listed in Table S1.

### WGA staining and immunofluorescence

Hearts were harvested and fixed in 4% paraformaldehyde, embedded in paraffin, and sliced into sections. The sections were dewaxed and incubated in water at 80 °C for 20 min. After cooling, the sections were washed, stained with wheat germ agglutinin (WGA), and subsequently incubated at 37 °C for 10 min. After the section were washed, they were sealed with glycerol and finally observed and imaged.

For immunofluorescence, the heart tissue was fixed, embedded, and sliced as described above. After the tissue sections were washed, they were incubated with anti-collagen I (1:200, Affinity, Cat: AF0134), anti-α-SMA (1:200, Affinity, Cat: BF9212), and anti-osteopontin (OPN) (1:200, Affinity, Cat: BF0002) primary antibodies and a fluorescently labeled secondary antibody before being observed and imaged.

### Luciferase reporter assay

We assayed the transcriptional activity of SOX9 by a luciferase reporter assay. The pGL3-SOX9-Luc reporter separately contained 12 repeats of a 48‐bp Col2a1 intron 1 enhancer element, which is known to be activated by SOX9, and the pRL-TK reporter was used as a control reporter. HEK-293T cells were cultured in 24-well plates and then transfected with 600 ng of pGL3- SOX9-Luc and 30 ng of pRL-TK. After 48 h, the cells were lysed, and the luciferase activity was measured with a Cytation 5 Cell Imaging Multi-Mode Reader.

### Lentiviral transfection

Lentiviruses were synthesized and purchased from GenePharma (Shanghai, China). The viral stocks were titrated by transduction of cells to 1 × 10^7^ transduction units (TU)/mL. For cell transfection, cells were transfected with siRNA lentivirus or a control lentivirus in 5 μg/mL polybrene. After transfection, the cells were used for intravenous injection or in vitro experiments.

### Statistical analysis

Bioinformatics data were analyzed using the R program (3.6.2, Austria) with the Bioconductor packages described above. GraphPad Prism 7.0 (CA, USA) was used for statistical analysis. The data are presented as the mean ± SD (for in vivo experiments) and were analyzed by *t*-tests, one-way ANOVA (for more than 2 groups) or two-way ANOVA (for more than 2 factors). A post hoc Tukey’s HSD test (for one-way ANOVA) or a Sidak HSD post hoc test (for two-way ANOVA) was performed when the ANOVA results showed significant interactions between variables. Repeated measures ANOVA was performed for time series data, and correlations were evaluated by Pearson’s test. A *P*-value < 0.05 was considered to indicate statistical significance.

## Supplementary information

Supplement Figure

Table S1

## Data Availability

Data from GEO database (GSE145477 and GSE145477), and Array express (E-MTAB7869) were used for analysis in this study.
