# Supplementary material for: G-MDSCs promote aging-related cardiac fibrosis by activating myofibroblasts and preventing senescence
Source: Cell Death Dis. 2021 Jun 8;12(6):594. doi: 10.1038/s41419-021-03874-7 (PMC8187421; doi:10.1038/s41419-021-03874-7)
Supplement: Supplementary file 1 — Supplement Figure [file 41419_2021_3874_MOESM1_ESM.docx]

**
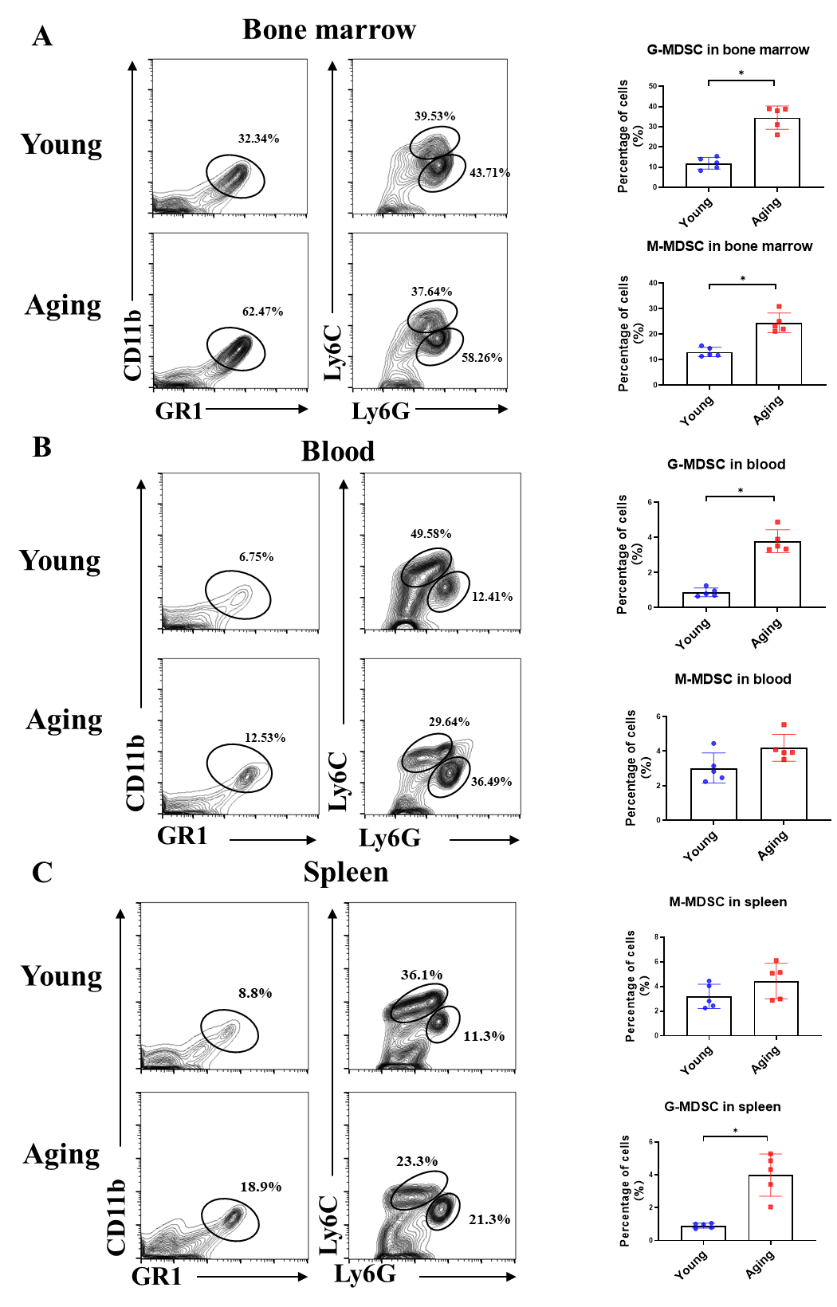
**

**Supplement Figure 1.** Representative flow cytometric profiles and statistical data showing the quantitative analysis of Cd11b+Gr1+Ly6G+ cells and Cd11b+Gr1+Ly6C+ cells in **(A)** bone marrow, **(B)** blood, and **(C)** spleen of young and aging mice; n = 5 per group. The data are presented as the means ± SDs. Differences were determined by Student’s t test. *P<0.05.

**
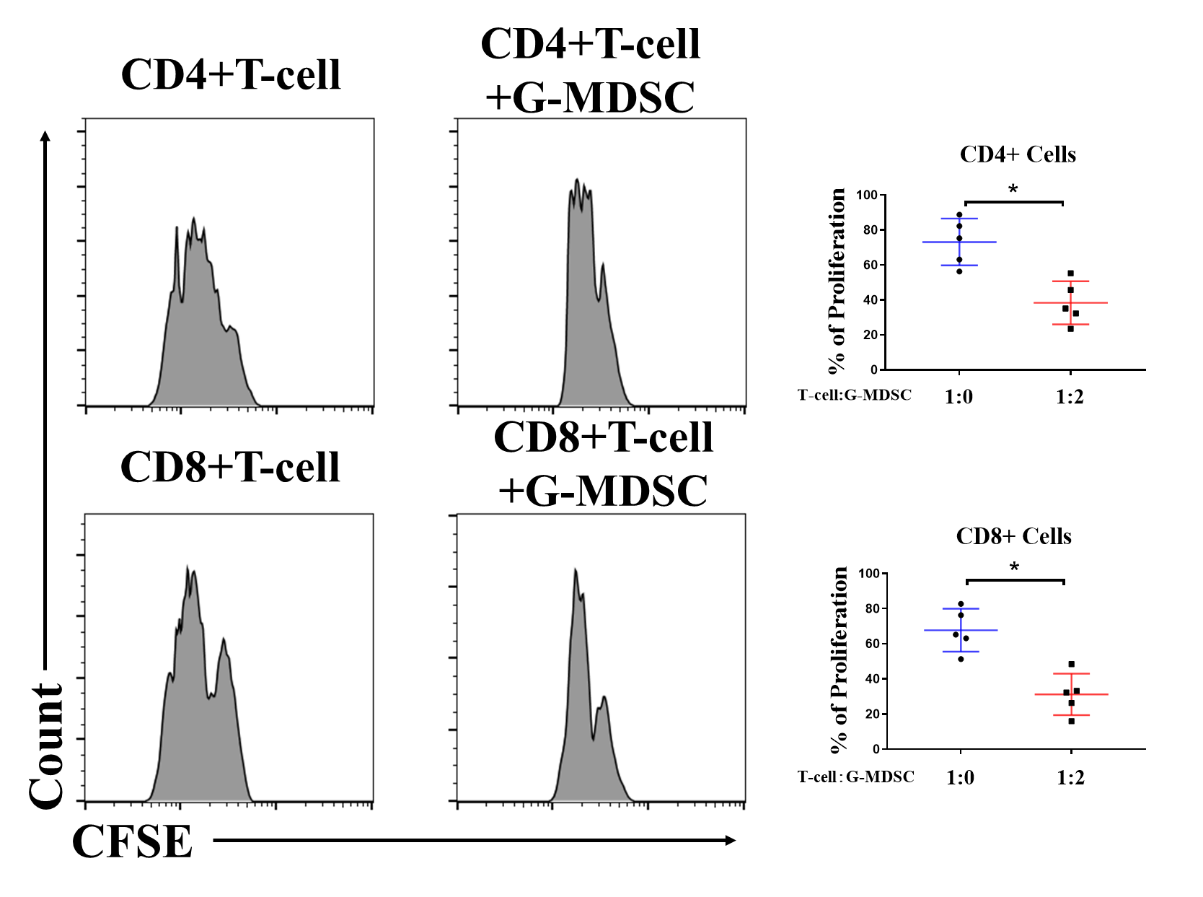
**

**Supplement Figure 2**. CFSE-labeled CD4+ and CD8+ T cells and MDSCs separated from spleens were cocultured at a ratio of 2:1 for 24 h. Representative images and quantitative analysis of the proliferation of CD4+ or CD8+ T cells analyzed by flow cytometry; n = 5 per group. The data are presented as the means ± SDs. Differences were determined by Student’s t test. *P<0.05.

**
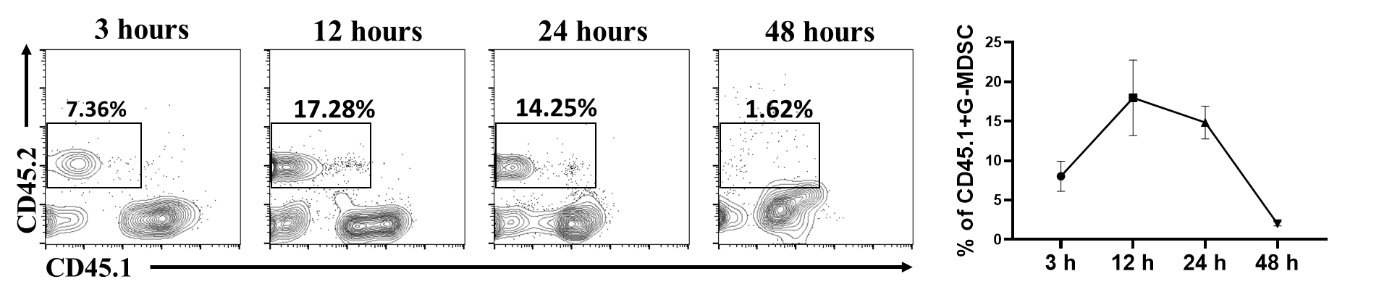
**

**Supplement Figure 3.** Representative flow cytometric profiles and statistical data showing the quantitative analysis of G-MDSCs of mice heart in recipient mice after adoptive transfer for 3 h, 12 h, 24 h, and 48 h; n = 8 per group.


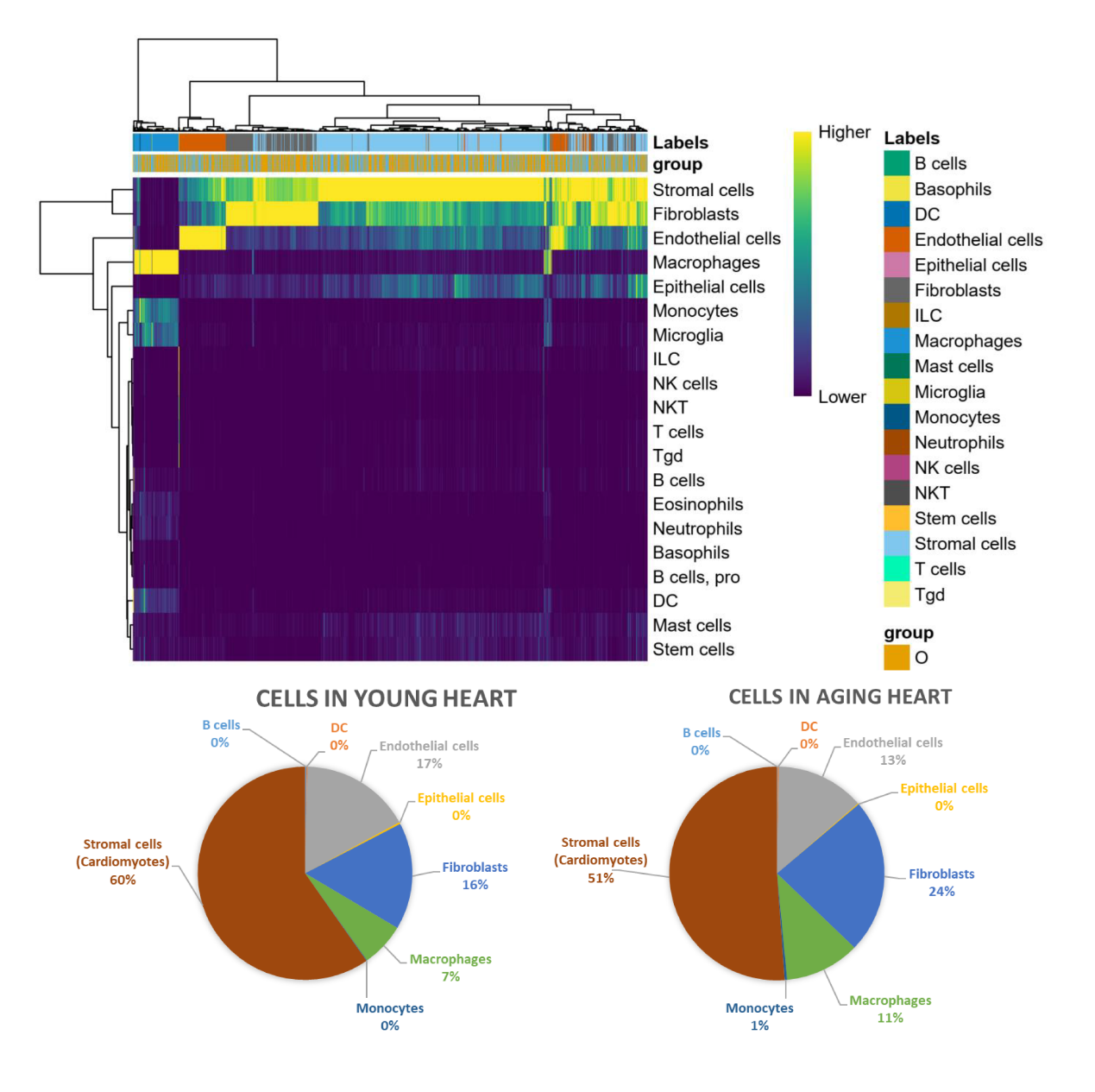


**Supplement** **Figure 4.** Heatmaps showing the distribution of various types of cells in young hearts and aging hearts (top). Pie charts showing the percentage of various types of cells in young hearts and aging hearts (bottom). The analyses were based on the scRNA-seq data ((E-MTAB7869).

**Supplement Figure 5.** G-MDSCs from aging hearts change fibroblast phenotypes. Fibroblasts were cocultured with G-MDSCs from young and aging mice. The mRNA levels of inflammatory factors (Il6, Tnf) and fibrotic markers (Acta2, Spp1, Fgf2) in fibroblasts were analyzed by qPCR; n = 5 per group. The data are presented as the means ± SDs. Differences were determined by one-way ANOVA (more than 2 groups), and Tukey’s HSD post hoc test was performed. *P<0.05.

**
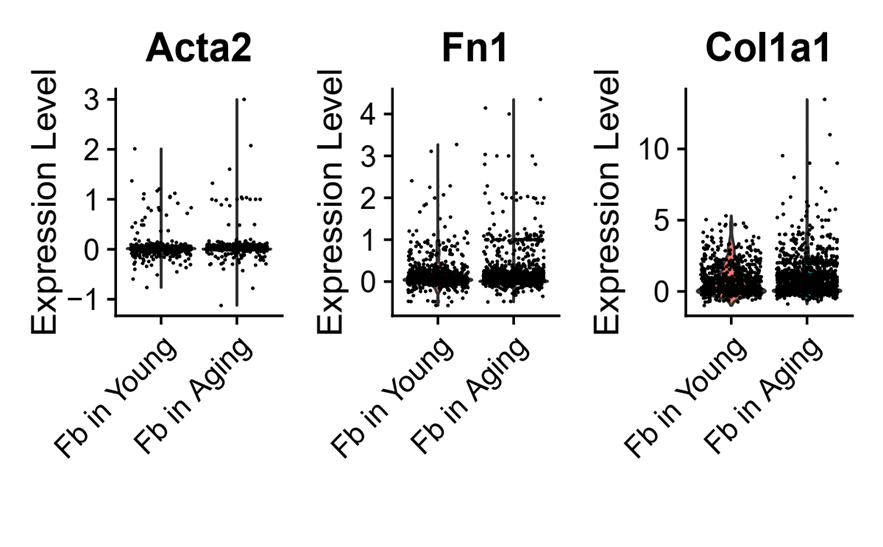
**

**Supplement Figure 6** The levels of fibrosis markers (Acta2, Fn1, Col1a1) in fibroblasts from aging hearts and young hearts. The analyses were based on scRNA-seq data (E-MTAB7869).


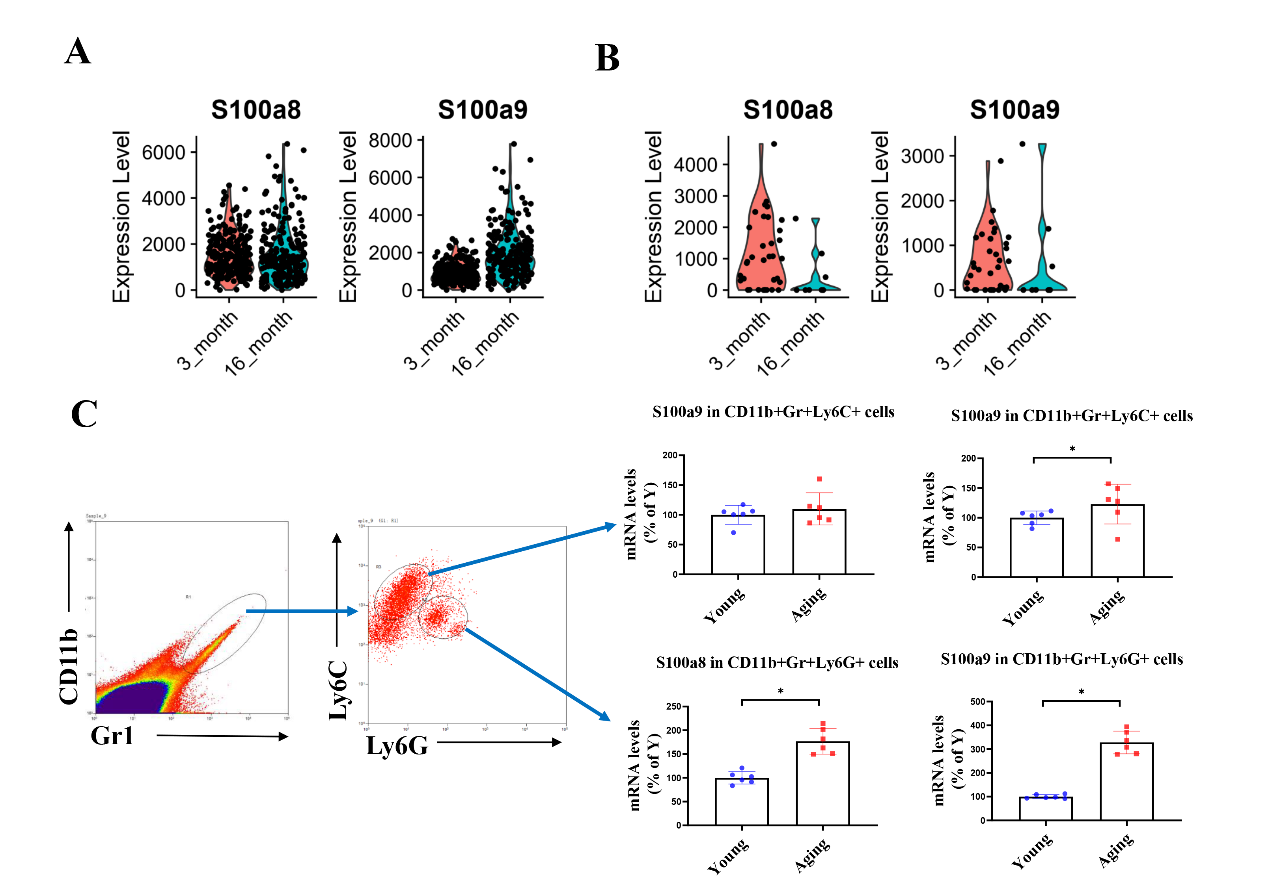


**Supplement Figure 7.** S100A8/9 mRNA expression levels were higher in G-MDSCs than in M-MDSCs. **(A)** The expression level of S100A8/9 in G-MDSCs from young (3-month-old) and aging (16-month-old) mice based on scRNA-seq data (E-MTAB7869). **(B)** The expression level of S100A8/9 in M-MDSCs of young (3-month-old) and aging (16-month-old) mice based on scRNA-seq data (E-MTAB7869). **(C)** The mRNA level of S100A8/9 in CD11b+GR1+Ly6C+ cells (M-MDSCs) and CD11b+GR1+Ly6G+ cells (G-MDSCs), analyzed by qPCR; n = 6 per group. The data are presented as the means ± SDs. Differences were determined by Student’s t test. *P<0.05.

**
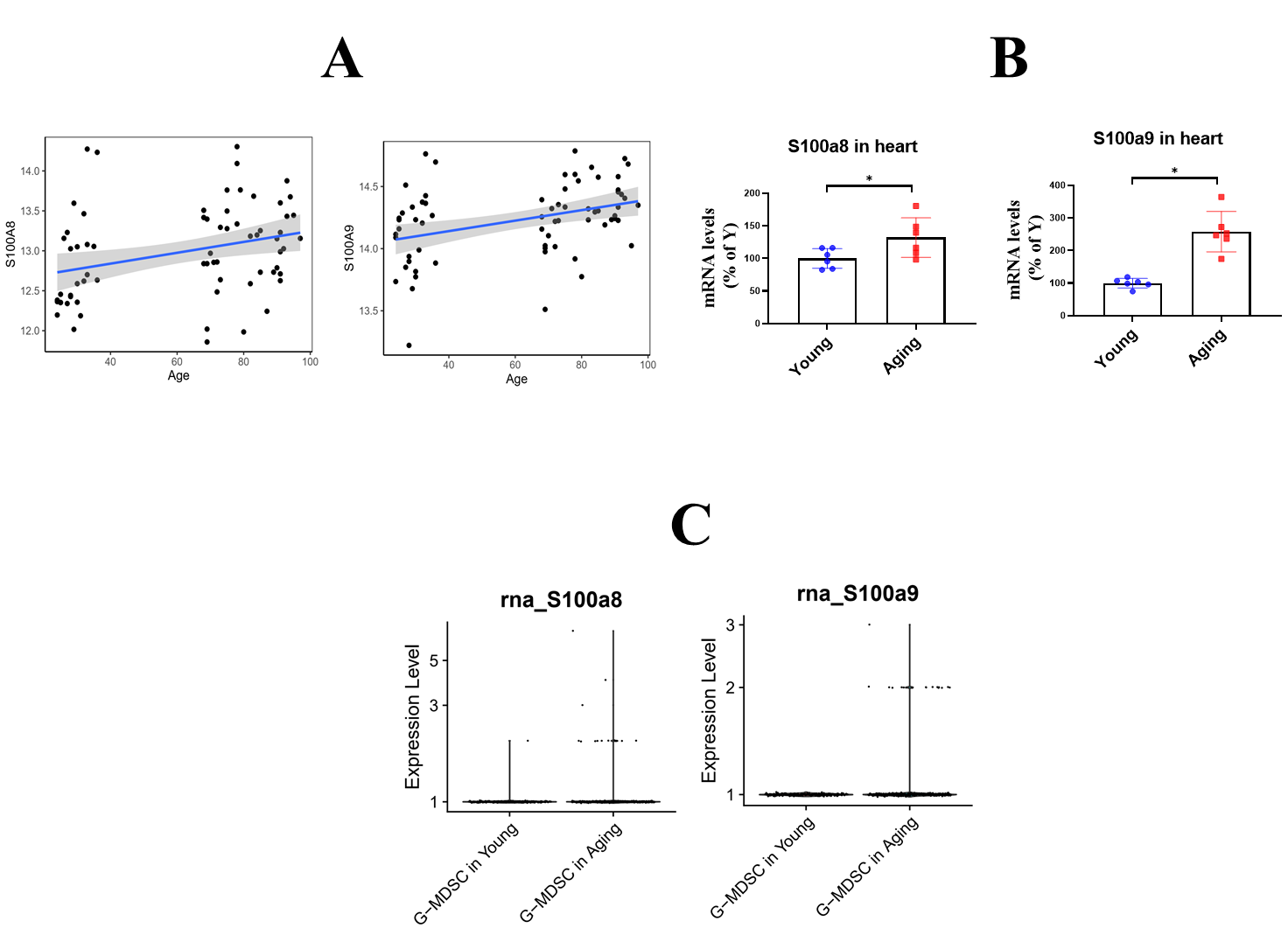
**

**Supplement Figure 8.** S100A8/9 expression levels in cardiac granulocyte-like cells (CD11b+Gr1+Ly6G+) were higher in aging mice than in young mice, and S100A9 expression increased significantly. **(A)** The bubble plots show the positive correlation between the mRNA levels of S100A8/9 and age in human peripheral blood leucocytes. The analyses were based on GEO data (GSE123698). **(B)** The mRNA levels of S100A8/9 in heart tissue of young and aging mice, analyzed by qPCR; n = 6 per group. **(C)** The expression levels of S100A8/9 in granulocyte-like cells of young and aging mice, analyzed through single-cell RNA-seq data (E-MTAB7869). The data are presented as the means ± SDs. Differences were determined by Student’s t test. *P<0.05.

**
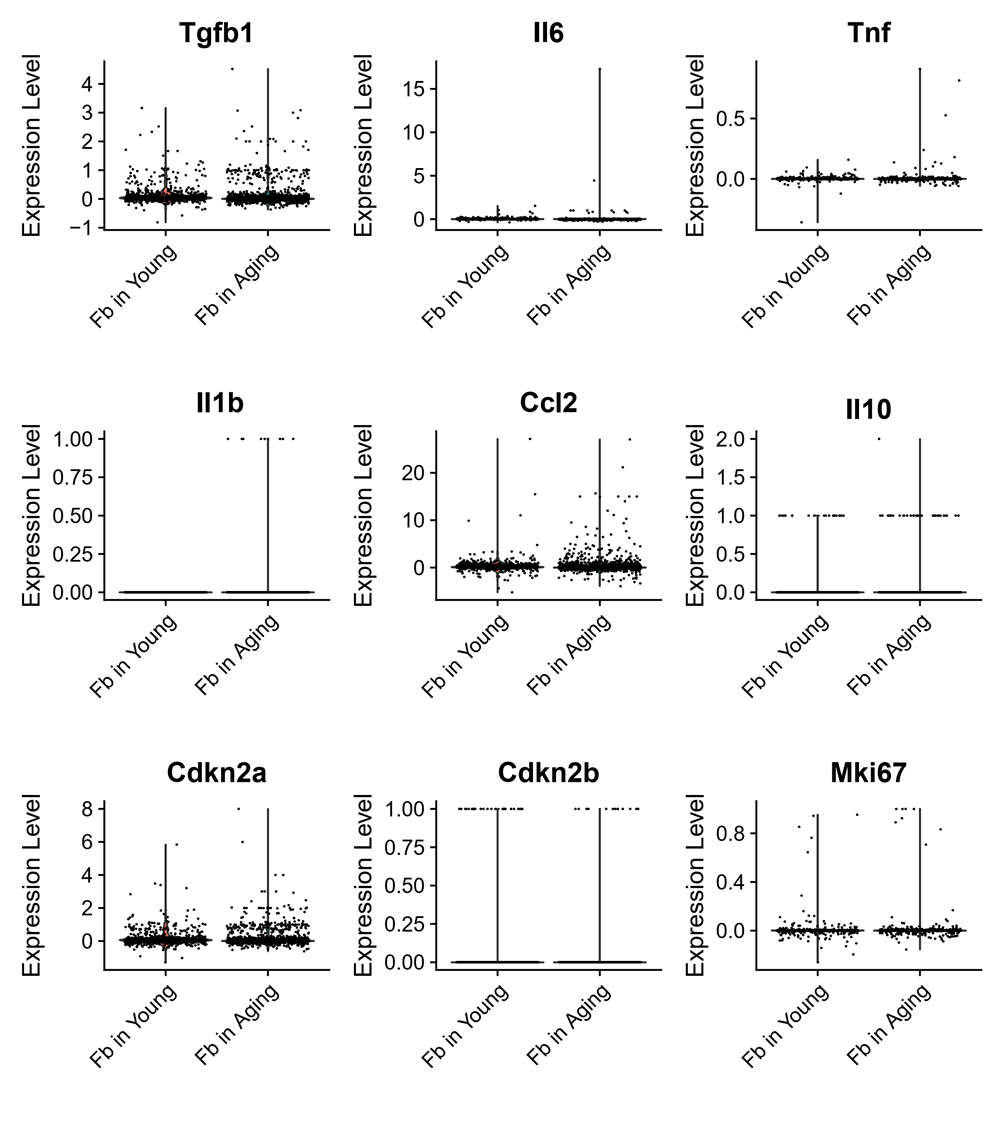
**

**Supplement Figure 9** The levels of Tgf-β, inflammatory cytokines (Tnf, Il6, Il10, Il1b) and senescence markers (Cdkn2a, Cdkn2b, Ccl2, Mki67) in fibroblasts from aging hearts and young hearts. The analyses were based on scRNA-seq data (E-MTAB7869).


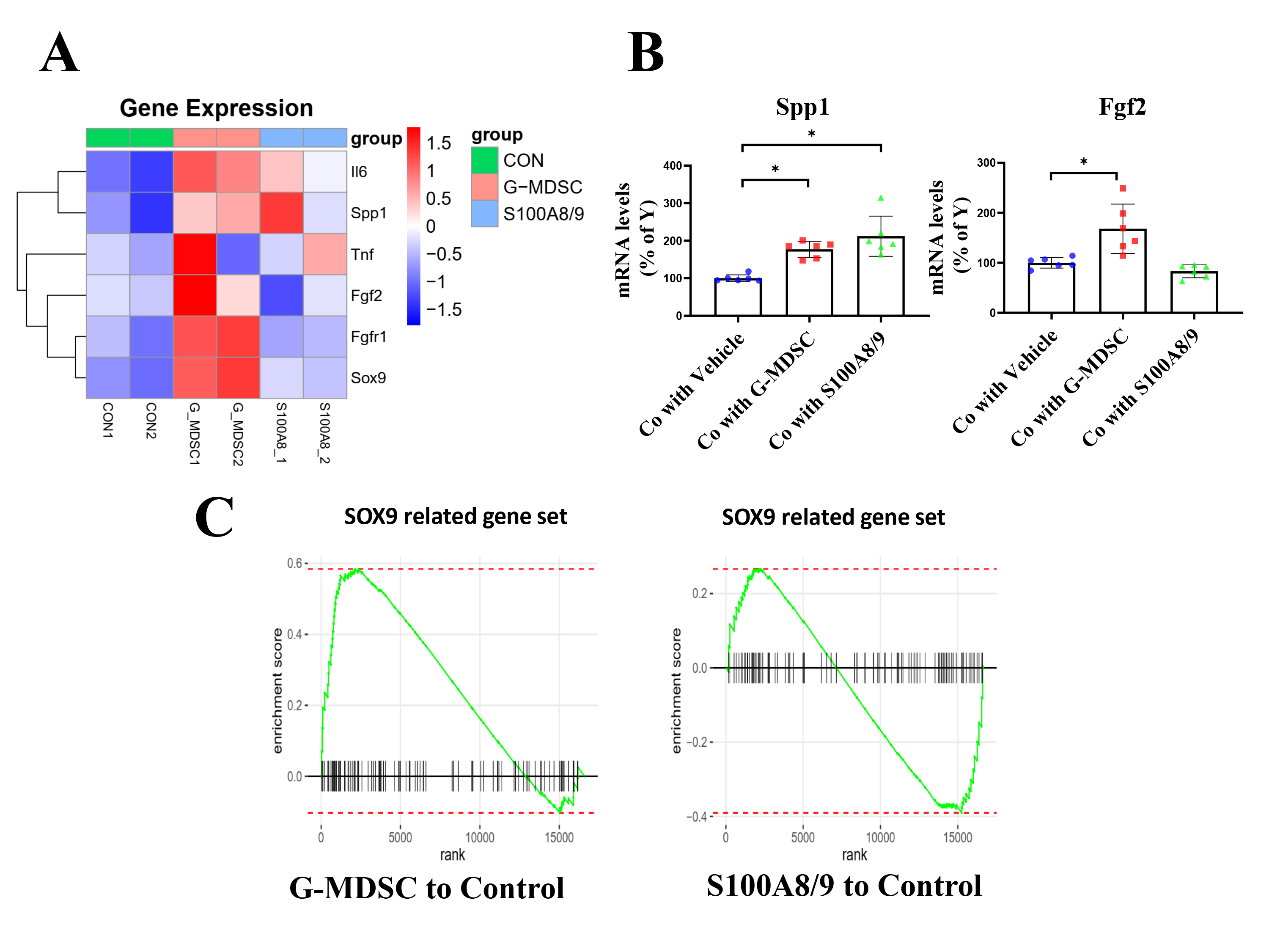


**Supplement Figure 10.** The S100A8/9-induced increase in OPN is independent of FGF2-SOX9 signaling. Fibroblasts were cocultured with G-MDSCs and S100A8/9. **(A)** Heatmap showing the gene expression level in the control group, G-MDSC group, and S100A8/9 group. **(B)** The mRNA levels of Spp1 and Fgf2 in the control group, G-MDSC group, and S100A8/9 group, analyzed by qPCR; n = 6 per group. **(C)** GSEA showing the SOX9-related gene set enrichment score in the G-MDSC group and S100A8/9 group. The data are presented as the means ± SDs. Differences were determined by one-way ANOVA (more than 2 groups), and Tukey’s HSD post hoc test was performed. *P<0.05.

**Supplement Figure 11** The mRNA levels of inflammatory cytokines (Tnf, Il6, Il10) in fibroblasts analyzed by qPCR; n = 5 per group. Differences were determined by one-way ANOVA (more than 2 groups), and Tukey’s HSD post hoc test was performed. *P<0.05.
