# Supplementary material for: G-MDSCs promote aging-related cardiac fibrosis by activating myofibroblasts and preventing senescence
Source: Cell Death Dis. 2021 Jun 8;12(6):594. doi: 10.1038/s41419-021-03874-7 (PMC8187421; doi:10.1038/s41419-021-03874-7)
Supplement: Supplementary file 2 — Table S1 [file 41419_2021_3874_MOESM2_ESM.docx]

Table S1 qRT-PCR primers for analysis of transcript levels

| Gene symbol | Forward5’-3’ | Reverse5’-3’ |
| --- | --- | --- |
| Col1a1 | ATCGGTCATGCTCTCTCCAAACCA | ACTGCAACATGGAGACAGGTCAGA |
| Col3a1 | CTGTAACATGGAAACTGGGGAAA | CCATAGCTGAACTGAAAACCACC |
| Postn | CACGGCATGGTTATTCCTTCA | TCAGGACACGGTCAATGACAT |
| Acta2 | CCCAGACATCAGGGAGTAATGG | TCTATCGGATACTTCAGCGTCA |
| Mmp2 | GTGTTCTTCGCAGGGAATGAG | GATGCTTCCAAACTTCACGCT |
| Mmp9 | GCAGAGGCATACTTGTACCG | TGATGTTATGATGGTCCCACTTG |
| Timp1 | CGAGACCACCTTATACCAGCG | ATGACTGGGGTGTAGGCGTA |
| Lox | CAGCCACATAGATCGCATGGT | GCCGTATCCAGGTCGGTTC |
| Lgals3 | GGAGAGGGAATGATGTTGCCT | TCCTGCTTCGTGTTACACACA |
| Tcf21 | CTCCCTGAAAGTGGACTCCAA | CGGGCTTTTCTTAGTGGGC |
| Il6 | CTGCAAGAGACTTCCATCCAG | AGTGGTATAGACAGGTCTGTTGG |
| Il10 | CTTACTGACTGGCATGAGGATCA | GCAGCTCTAGGAGCATGTGG |
| Il1b | GAAATGCCACCTTTTGACAGTG | TGGATGCTCTCATCAGGACAG |
| Tgfb1 | CCACCTGCAAGACCATCGAC | CTGGCGAGCCTTAGTTTGGAC |
| Cdkn2a | CGCAGGTTCTTGGTCACTGT | TGTTCACGAAAGCCAGAGCG |
| Cdkn2b | CCCTGCCACCCTTACCAGA | GCAGATACCTCGCAATGTCAC |
| Tnf | GGTGCCTATGTCTCAGCCTCTTTT | GCCATAGAACTGATGAGAGGGAG |
